# Correction: A mindfulness-based stress management program for caregivers of allogeneic hematopoietic stem cell transplant (HCT) patients: Protocol for a randomized controlled trial

**DOI:** 10.1371/journal.pone.0275398

**Published:** 2022-09-23

**Authors:** Min-Jeong Yang, Valerie V. Yepez, Karen O. Brandon, Maija Reblin, Joseph Pidala, Heather S. L. Jim, Jerrold S. Meyer, L. Robert Gore, Nandita Khera, Penny Lau, Rachel M. Sauls, Sarah R. Jones, Christine Vinci

## Notice of republication

In light of an issue identified post-publication, this article was republished on September 13, 2022, to replace S1 File and remove information that was not relevant to the article. Please download this article again to view the correct version. The originally published, uncorrected article and the republished, corrected articles are provided here for reference.

## Supporting information

S1 File Originally published, uncorrected article. (PDF)

S2 File Republished, corrected article. (PDF)
