# Supplementary material for: A mindfulness-based stress management program for caregivers of allogeneic hematopoietic stem cell transplant (HCT) patients: Protocol for a randomized controlled trial
Source: PLoS One. 2022 Apr 1;17(4):e0266316. doi: 10.1371/journal.pone.0266316 (PMC8975158; doi:10.1371/journal.pone.0266316)
Supplement: S1 File — (DOCX) [file pone.0266316.s002.docx]

## **Study Protocol**

Allogeneic hematopoietic stem cell transplants (HCT) are a possibly curative treatment for hematologic malignancies/disorders. However, these transplants are extremely intensive and require a full-time caregiver.1 Caring for an allogeneic HCT patient is a significant responsibility, involving a commitment to be available 24/7 up to at least 100 days post-transplant to provide emotional and physical support.2,3 Throughout the cancer care continuum, informal family caregivers of HCT patients often experience significant role changes that can conflict with other responsibilities (e.g., work, child rearing), and caregivers often report high burden and poor mental health.4-6 Caregiver burden and distress can have an adverse impact on patient health outcomes, including increased anxiety, depression, and decreased patient survival.7-12 However, some research suggests that caregivers may benefit when they receive adequate support and when given the opportunity for growth and meaning-making.12-15 Despite this, few comprehensive and proactive caregiver support programs exist.16

Mindfulness facilitates the ability to shift attention to experiences purposefully, with a sense of acceptance (as opposed to trying to change or react to it).17 Active mechanisms of mindfulness (e.g., improved attention, emotion regulation, decentering)18-22 combined with the unique circumstances of HCT caregivers, suggest that approaching thoughts/emotions/sensations via mindfulness may convey benefits above and beyond what is found in other treatment approaches.23-25 Mindfulness has been associated with psychosocial benefits in other populations (e.g., decreased negative affect26-31 and stress32,33). To date, a few small-scale, single-arm studies have investigated mindfulness for the cancer patient-caregiver dyad with promising results.34-38 There have been no studies examining mindfulness for HCT cancer caregivers. Our multi-disciplinary team systematically developed a 6-week mindfulness-based intervention for allogeneic HCT caregivers – FOCUS (**F**ocusing **O**n mindfulness for **C**aregivers **U**nder **S**tress) – with input from caregivers and transplant team members. FOCUS spans the course of patient treatment, with session 1 occurring prior to transplant, sessions 2-3 taking place immediately following transplant, and sessions 4-6 occurring post-discharge. Pilot testing revealed that FOCUS was highly feasible and acceptable for allogeneic HCT caregivers and useful for managing stress.39 Significant decreases in negative affect and increases in mindfulness, post traumatic growth, and mental health symptoms were observed from baseline to end of treatment; findings were maintained through the 1-month follow-up.

This project will build on our pilot study by using a 1:1:1 randomization of caregivers (N=270) to one of three treatment conditions: FOCUS, Healthy Living (HL), and Usual Care (UC). This three-arm design will allow us to rigorously test if FOCUS is more efficacious than both HL (attention placebo condition matched to FOCUS on time and contact) and UC (attention control condition). Self-report assessments will occur at baseline, end of treatment, and 2- and 6-months post-treatment (to determine if initial treatment gains remain over time). In addition, biomarker data will be collected via hair cortisol concentrations, and daily diaries will assess fluctuating variables (e.g., affect, state mindfulness) among all three conditions. Biomarker and daily diary data are novel aspects of this project that permit a more thorough investigation of mechanistic variables. We anticipate mindfulness to ultimately reduce caregiver burden (primary outcome) via measured mechanisms (e.g., reduced stress). We expect patient distress and healthcare utilization to be both directly and indirectly impacted via caregiver participation in FOCUS.

### Aim 1. Evaluate the efficacy of FOCUS when compared to UC and HL on caregiver burden.

H1a and H1b: Caregivers in FOCUS will report lower levels of burden 2 months post-treatment than caregivers in UC (H1a) and HL (H1b). Secondary outcomes include depression, anxiety, and post traumatic growth.

### Aim 2. Evaluate the impact of FOCUS on patient health and patient healthcare utilization.

H2a and H2b: Patients of caregivers in FOCUS will report lower levels of distress 2 months post-treatment than patients of caregivers in UC (H2a) or HL (H2b).

Exploratory: Determine the impact of FOCUS on patient healthcare utilization in the first 2 months and from 3 to 6 months post-treatment (hospital readmissions, length of readmissions, unexpected patient clinic visits).

### Aim 3. Evaluate putative mechanisms and moderators of FOCUS.

We expect that caregivers in FOCUS will demonstrate higher mindfulness and self-efficacy, and lower stress (self-report and via hair cortisol) and negative affect as collected via daily diary and at the 2 month assessment than caregivers in UC and HL. Potential moderators of treatment on caregiver outcomes will be evaluated (e.g., gender, age, relationship to patient, income, proximity of home residence to cancer center).

This proposal aligns with a recent call from NIH to develop and test interventions for cancer caregivers that also improve patient health outcomes.40 If ultimately proven efficacious, future research includes plans for

dissemination/implementation, as well as extension to other cancer caregiver populations.

**Project Overview and Timeline**. Caregivers (N=270) will be randomized (1:1:1) to one of three study conditions: FOCUS, Healthy Living (HL), or Usual Care (UC). Both caregivers and patients will be assessed at baseline, end of treatment (~8 weeks post-baseline), and 2- and 6-months post-treatment. Figure 3 presents the study timeline for caregivers and patients, anchored by the day of the patient’s transplant (Day 0). Patients and caregivers will be recruited and consented about 2 weeks (~Day -14 to ~~ Day -10) prior to the patient’s scheduled transplant day. Patients are often in the clinic for vital organ testing in preparation for HCT at this time. The caregiver will then be randomized to FOCUS, HL, or UC. For FOCUS and HL, we plan for session 1 to occur the around week prior to transplant, session 2 the week of transplant, and session 3 the first week post-transplant. These sessions will be scheduled at the appointment that coincides with our timeline, but because there may be medically related changes in schedules (e.g., unanticipated complications), we will be flexible in the timeline for sessions. Sessions 4-6 will occur post-discharge, once the patient is released within a 30 minute radius of the cancer center (patients and caregivers typically stay within 30 minutes of the cancer center for the first 100 days post-transplant). Although this schedule will accommodate most patient/caregiver schedules, we expect some schedules to differ. For instance, some patients have their discharge around Day+18-22, if certain symptoms need to resolve. We encountered these issues during the pilot study and will flexibly accommodate such changes as they arise in the current study.


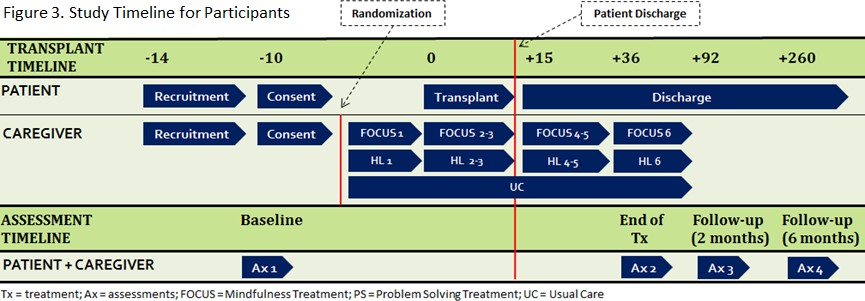
The end of treatment assessment will take place within approximately 1 week of session 6. Follow- up visits will occur about 2 months and 6 months post-

treatment. The 2- month follow-up time point will allow us to capture short- term treatment gains reported since the

end of treatment assessment. We anticipate very little missing data at this point, as the caregiver/patient are still required by the treatment team to be living within 30 minutes of the cancer center. By the 6-month follow- up, caregivers/patients have usually returned home, and we will be able to observe any long-term treatment gains at this time. Assessments will be sent to participants as needed. We may encounter more missing data at this time point, and our analytic plan outlines how this will be handled. All caregivers will be invited to complete daily diary questions via their smartphone during the six active weeks of FOCUS, HL, or UC (see Section C.5.5 for additional information).

**Participants***.* Although the patient-caregiver dyad will be recruited for this study, the intervention will be delivered only to caregivers. Caregivers will be randomized to one of three conditions and complete a total of 6 treatment sessions (if in FOCUS or HL) and a full set of questionnaires at each assessment. The patient will only complete a set of very brief questionnaires at each assessment. Inclusion/exclusion criteria will be driven by the caregiver (i.e., we will assess caregiver eligibility and if eligible, we will then assess patient eligibility). Caregiver inclusion/exclusion include: 21 years of age or older; caring for a patient planning to receive an allogeneic HCT at Moffitt; intending to remain primary caregiver throughout patient treatment (i.e., will be the caregiver the majority of the time); able to provide informed consent; able to read and write in English; and owns a smartphone and is willing to download the study app. Patient inclusion/exclusion criteria will include: 21 years of age or older; receiving an allogeneic HCT at the cancer center; able to provide informed consent; and able to read and write in English.

**Recruitment**. Recruitment will occur at Moffitt Cancer Center on the HCT unit, and our team has developed

successful recruitment and retention strategies in previous work,39,108-110 all while considering potential barriers in

recruiting certain cancer dyads for research.111-113 This includes reducing participant burden by tying our study

timeline to scheduled clinic visits and implementing web-based questionnaires and study tools. During the pilot

phase of the study, the recruitment method outlined below was very successful and ultimately allowed study

staff to contact each person who was admitted for a transplant. Over 4 years we anticipate consenting about 270

participants. When considering a dropout rate of 30%, that would leave 189 participants (n=63 per group) for

analysis.

**Initial Screening and Informed Consent**. Each week, study staff will receive a list of allogeneic patients who are scheduled to be admitted within the next 7-10 days. At this time, study staff will conduct a chart review to determine initial eligibility of the caregiver. Of those who are potentially eligible, the caregiver contract in the patient chart will be located and a phone call will be made to the caregiver directly to explain the goals of the study and further assess eligibility. If interested, study staff will schedule an in-person meeting to go over study details and complete the informed consent process; this visit may coincide with a patient visit. Patients of interested caregivers will be contacted either by phone or in person (at a clinic visit), prior to the caregiver informed consent session. Similarly, if interested, the patient will schedule their consent session to coincide with a clinic visit. In most cases, the consenting session for caregivers and patients will occur at the same clinic visit. During the pilot study, all caregivers were consented in clinic, coinciding with a patient visit. Study staff will consent each separately. Caregiver consent will occur first, and if the caregiver consents, the patient will be invited to complete the informed consent process. From there, if the patient declines to participate, the caregiver may still participate. We will also collaborate with social workers and other members of the treatment team in the HCT department, such that they can directly provide our study information to a caregiver, should the opportunity present itself. We will follow the procedures above for any caregiver referred from treatment team. Once consented, if either member of the dyad drops, the other will be allowed to remain in the study.

**Assessment Visits.** After informed consent, caregivers will complete a battery of baseline questionnaires and a hair sample will be collected for cortisol testing (100% of caregivers allowed us to take hair samples during the pilot study). We anticipate this process taking about 30-40 minutes for the caregivers to complete. After completing questionnaires, caregivers will schedule their 6 treatment visits. Patients will complete a brief demographic questionnaire and three measures to assess distress. This should take about 10 minutes for patients to complete.

The same assessments will be completed at baseline, end of treatment (~8 week post baseline), and at follow-ups. Hair samples will be collected again from caregivers at the 6-month assessment. Whenever possible, assessment visits will coincide with a clinic visit to decrease burden. As needed, questionnaires will be mailed, texted (link) or emailed to participants (e.g., 6-month visit). Caregivers will be compensated $25 for baseline, end of treatment, and the 2-month follow-up visits; they will be paid $50 for the 6-month follow-up. Patients will be compensated $20 at each assessment time point (4 total). As described in Section C.5.5., caregivers will be asked to complete a daily diary each evening that should take no more than 2 minutes to complete. Caregivers who complete at least 70% of daily diary questions will receive a bonus payment of $40 at the end of treatment assessment visit.

A subsample of patients will be asked to complete a telephone interview (approximately 30-45 minutes in length) in order to obtain feedback about the possibility of having patients engage in a stress management intervention in a future study. Approximately 30 patients whose caregivers were randomized to either the HL or FOCUS interventions will be asked to participate in the phone interviews at approximately the 2-4 month post EOT follow up period. Every other patient enrolled with a caregiver in one of those two conditions will be asked to participate in an individual phone interview. The number of patients asked to participate will be approximately 15 per caregiver’s condition (HL or FOCUS) or until saturation of feedback (i.e., no new information is offered by participants). Approximately 20 open ended questions will include queries about the patient’s emotional and physical experience around the transplant process, including the pre-hospitalization, hospitalization for transplant, and post-hospitalization periods. Specifically, stress, coping, stress management, and awareness of caregiver’s stress management program will be queried. Patients will also be asked for their thoughts about offering stress management to patients and what types of support would have been useful. Interviews will be recorded, transcribed, and coded for themes.

**Randomization.** Participants (caregiver/patient dyads) will be randomized at enrollment, to one of the three treatments using balanced-permuted blocks of size six toward assigning 90 dyads to each condition. Caregivers will be stratified by gender prior to randomization as research indicates male and female cancer caregivers differ on distress and coping styles.114

**Retention Procedures**. We will conduct the following procedures to reduce attrition: reminder phone calls and messages via phone app prior to all study visits; flexible scheduling of caregiver treatment sessions to accommodate different schedules; meeting with the caregiver for sessions 1-3 on the transplant unit; meeting for sessions 4-6 via video conference call; sending cards with thoughtful messages to patients and caregivers at some occasions that may occur during their time in the study (e.g., birthday, holiday, 4 month post treatment, etc.); providing small welcome or thank you gifts that may contain with the study logo (e.g., tote bag, pens, phone stand, etc.),.) and requiring a functioning phone number and home address to contact participants by phone or mail as needed.

### Treatment Overview.


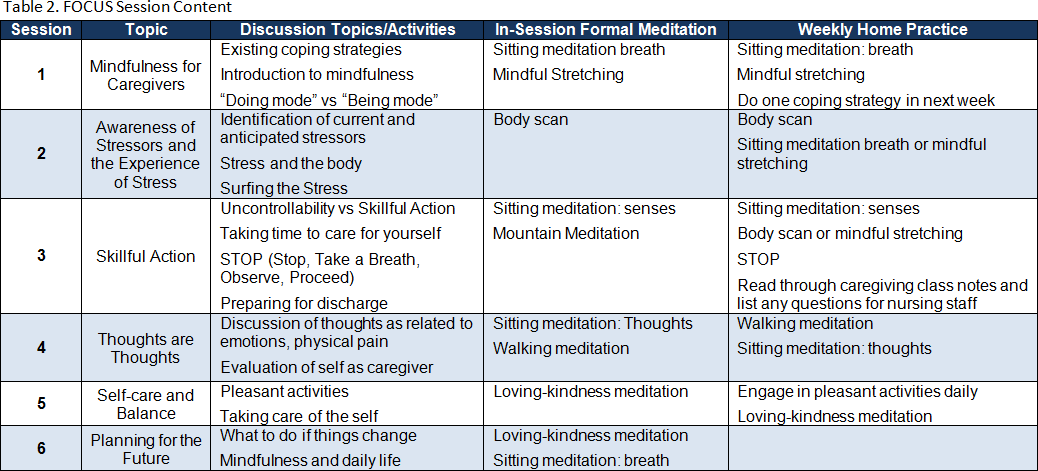
Participants in all treatment conditions will receive a list of support groups and contact information for social work at the baseline visit by study staff. All caregivers are required to attend a caregiver class, prior to patient discharge that outlines their responsibilities as a caregiver (e.g., meal prep, medication administration).**FOCUS**. FOCUS is a manualized treatment and is comprised of six one-on-one, 45-60 minute sessions delivered either in-person (sessions 1-3) or via video conference (sessions 4-6).39 To be flexible with caregiver schedules and COVID-related restrictions, treatment delivery in person vs zoom my vary as needed. As is typical in other MBIs, the first few sessions primarily focus on how to direct attention to the breath or some object of attention (e.g., parts of the body). As the program progresses, participants are asked to apply these skills to thoughts and emotions. Throughout the treatment, caregivers are reminded to utilize existing coping skills, as well as how to integrate the new skills learned throughout this program for managing stress. Formal mindfulness meditations are conducted within each session, lasting from 7-20 minutes; participants are asked to practice

mindfulness exercises daily. Table 2 provides a brief overview of each session’s content, formal meditation practice, and home practice. At the end of session 3, caregivers are asked to consider how to prepare for the discharge of the patient, as this is the usual timeframe for most patients to be

discharged. However, some patients are discharged at a later date due to medical complications. In these cases, the session content on “preparing for discharge” will be moved as needed. All participants will receive a booklet (created and used during pilot) that includes session content for each week.

In order to and facilitate compliance with treatment, we will ask participants to download a phone application that facilitates some or all of following: meditation recordings, video conference for sessions 4-6, session reminders, and daily meditation reminders. To fully leverage the phone app and better facilitate the integration of mindfulness into daily life, we will also send participants very brief mindfulness strategies to practice throughout the day (1-3 per day), favoring brief interpretations of mindfulness exercises that might be particularly useful to caregivers (e.g., “Notice any thoughts you’re having in this moment, and watch them go by as if they are clouds in the sky” or “If possible, let go of any judgments that are good or bad right now. Every moment is new, so focus on just this moment.”). Content areas of the messages include: focusing on the breath, noticing thoughts, awareness of sensations, motivational messages, acceptance/non-judgment., gratitude, lovingkindess, and uncertainty. Providing these strategies to caregivers throughout the day should further enhance and reinforce what they are learning during the in-person sessions.

**Healthy Living (HL).** The HL intervention will match FOCUS in time and provider contact (i.e., six, 45-60 minute sessions delivered one-on-one; sessions 1-3 in-person on HCT unit and sessions 4-6 via video conference). To be flexible with caregiver schedules and COVID-related restrictions, treatment delivery in person vs zoom my vary as needed. HL will be based on the American Cancer Society’s (ACS) *Caregiver Resource Guide.^115^* We will specifically focus on those sections most relevant to this population (e.g., Cancer Information and Caregiver Self-Care). A booklet will be created for HL and will only contain the topics presented below. We specifically chose topics that are not teaching active coping strategies to manage stress. Instead, we included information on general topics of healthy living that are commonly known and available to the public (e.g., recommended servings of daily fruits and vegetables). Thus, we do not anticipate HL to have a direct or indirect impact on primary outcomes (e.g., burden), or active mechanisms (e.g., reduced stress).


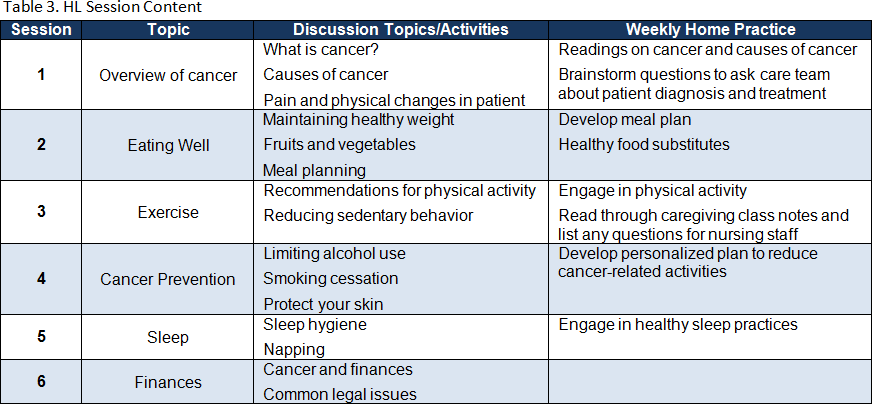
Table 3 outlines specific topics that will be discussed each week. Session 1 will

present an overview of cancer, including origins of cancer, common causes of cancer, and what to expect regarding pain and physical changes in the patient.

Session 2 will focus on healthy eating (e.g., recommendations for healthy eating, meal planning). Session 3 will provide recommendations for physical activity and reducing sedentary

behavior. Session 4 will discuss general cancer prevention recommendations regarding alcohol use, tobacco, and skin protection. Session 5 will focus on basic sleep hygiene (e.g., turning off TV prior to bed, limiting alcohol use before bedtime). Session 6 will discuss the impact of cancer on finances. Home practice each week will be directly related to the in-session topic.

Participants will also use the phone app, which will allow both FOCUS and HL to match on technology interaction. Identical features of FOCUS and HL include video conference for sessions 4-6 and session reminders. Participants in HL will have access to static content on the app related to the session content (e.g., standard recommendations for physical activity) that will become available after the session it is introduced. In order to match the FOCUS intervention on number of contacts, the HL participants will also receive, via the app, brief messages that remind them about the reminders to content for the week (e.g., “Getting exercise may help reduce your stress”) and motivational messages. The number of these messages will match the FOCUS group.

**Usual Care (UC).** Participants in UC will receive treatment consistent with what is offered to all caregivers of allogeneic HCT patients. This entails the option of attending weekly support groups and meeting with social workers as needed. At the baseline session, participants randomized to UC will be provided with a modified version of the ACS *Caregiver Resource Guide* by study staff.115 This booklet will be identical to the booklet given to caregivers in HL. Ethically, we believe providing some additional information to this group is warranted, given they are agreeing to be part of a research study during an incredibly difficult time in their lives.

**Treatment Delivery and Fidelity.** Explicit facilitator selection criteria, extensive training, and on- going monitoring and supervision of treatment delivery, fidelity, and facilitator competence will ensure that treatments are of the highest quality, follow the protocols precisely, and prevent counselor drift and contamination. Given the substantial differences between FOCUS and HL, and to reduce any therapist-specific effects, facilitators may concurrently provide both treatments. We anticipate the likelihood of treatment crossover and subsequent treatment contamination to be very minimal.

*Selection Criteria.* Facilitators must have experience in counseling, psychology, social work, or a related field. Given FOCUS is a MBI, facilitators also need to have or complete mindfulness training, and have a daily formal and informal mindfulness practice.

*Training, Supervision, Adherence*. Facilitator training for FOCUS and HL will be conducted by Dr. Vinci, as she is a clinical psychologist with extensive experience in delivering behavioral treatments; training and supervision may also take place with or another individual with similar mindfulness training and experience. Facilitator training will include readings of the FOCUS and HL manuals, practicing and role playing each session of FOCUS and HL, and working through anticipated issues that might arise during sessions. Training will occur in a regular series of half-day blocks (1-2 per week) with the facilitator studying and role-playing between training sessions. Training will continue until the facilitator reaches performance criteria for competence and adherence to the protocols, which will be made on facilitator rating forms during mock sessions using a modified version of the validated Mindfulness-Based Relapse Prevention Adherence and Competence Scale (MBRP-AC)116 for FOCUS and a measure created for HL for the current study. To ensure fidelity to treatments once participants are enrolled in the study, regular supervision with facilitators will be conducted to review session recordings, discuss miscellaneous counseling issues, and problem-solve as needed. These supervision sessions will ensure that each treatment is being delivered with fidelity.

*Assessment of Treatment Fidelity*. To monitor facilitator adherence and competence, all sessions will be audio-recorded and a random sample of 10% will be rated by the investigators using the MBRP-AC for FOCUS and a measure created for HL for the current study. Home practice will be monitored weekly via phone app and at the end of treatment assessment to measure treatment adherence (e.g., amount of time each day spent meditating; use of self-care). Finally, at the end of treatment assessment, caregivers will complete a measure to assess facilitator characteristics (e.g., warmth, empathy).

**Measures**. Several major considerations guided our measurement selection. First, assessment selection criteria included established reliability and validity. Second, assessments had to either a) represent hypothesized treatment mechanisms/effects or b) have been empirically relevant to our population. Third, to reduce the inconvenience associated with completing the assessments, we will provide compensation for participants’ time. A summary of all measures is provided in Table 4, including the number of items per measure.

**Demographics and Caregiver/Patient Characteristics**. Demographic information will be collected at baseline from both caregivers and patients and will include gender, age, ethnicity, race, partner status, income, and education. Additional questions for the caregiver will include relationship to patient and proximity of home residence to the cancer center. Attendance at any support groups or meetings with social workers by caregivers will also be collected at each assessment visit. Clinical characteristics (e.g., cancer type, treatment) will be assessed by chart abstraction.

**Aim 1 Caregiver Outcomes**. The primary caregiver outcome, burden, will be assessed by the Zarit Burden Interview Short Form (ZBI), which uses a 5-point Likert scale.117 The ZBI has been validated in populations of cancer caregivers and has demonstrated very good internal consistency and discriminative ability.118 This measure has also been used to identify changes over time.119

Secondary outcomes will assess several constructs relevant to the HCT caregiver. The Center for Epidemiologic Studies Depression Scale (CESD) will assess depression on a 4-point Likert scale120 and has been commonly used among cancer caregivers.4,121 The Generalized Anxiety Disorder-7 (GAD-7) will measure anxiety among caregivers on a 4-point Likert scale122 and has been used extensively among cancer caregivers.123,124 The Post Traumatic Growth Inventory (PTGI) will evaluate the caregiver’s assessment of any positive outcomes that may have been due to the patient receiving a diagnosis and treatment for cancer on a 6-point Likert scale.125 The ability to find benefit from a stressful life experience is associated with increased resiliency and personal satisfaction.13,14 We expect FOCUS to impact benefit finding/post traumatic growth, given post traumatic growth is associated with the ability to reappraise/reframe stressful situations,42,43 and nonjudgmental awareness taught via mindfulness should aid in the ability to reframe a given situation.52-54

**Aim 2 Patient Outcomes**. Patient distress will be captured with CESD120 to measure depression and the GAD-7 to measure anxiety.122 The CESD is commonly used among cancer patients with strong reliability and validity estimates;126,127 it has been used extensively among MBIs for cancer patients.128 Given the CESD does not include items indicative of cancer symptoms (e.g., weight loss, fatigue) that may overlap with depression, it is an ideal measure to use in this population. Similarly, the GAD-7 is a commonly used measure among cancer patient populations that has also been utilized within MBIs for cancer patients.123,129,130

**Aim 2 Patient Healthcare Utilization Outcomes**. Healthcare utilization will be assessed by extracting the following from patient charts: readmissions to the hospital, length of stay of all hospital readmissions, and unexpected clinic visits post-discharge. Unexpected clinic visits post-discharge will be a secondary measure of healthcare utilization. These visits will be captured when patients are scheduled as a “triage” visit, as all triage visits are considered unexpected when documented in this manner in the clinic. At the 6 month follow-up assessment, healthcare utilization outcomes will be extracted from the chart when possible, but self-report and supplementation with outside records will also be done in cases when the caregiver/patient is no longer being seen at Moffitt (e.g., moved back home).

Additional Caregiver Variables (mechanisms, moderators, possible covariates). Stress and affect will be captured by self-report and biomarker data (cortisol). The Perceived Stress Scale (PSS) assesses one’s perception of life as unpredictable, uncontrollable, and overwhelming on a 5-point Likert scale.131 The Impact of Events Scale Revised (IES) assesses intrusion, arousal, and avoidance symptoms around the diagnosis of cancer on a 5-point Likert scale. The Positive and Negative Affect Schedule (PANAS) assesses broad levels of positive and negative affect (e.g., determined, nervous) on a 5-point Likert scale.136The National Comprehensive Cancer Network Distress Thermometer will be used as a 1-item measure of overall (non-specific) distress in the past week.

Hair samples will be collected at baseline and the 6 month follow-up to measure cortisol concentrations from the prior month.137-139 Our consultant, an expert in the measurement of hair cortisol concentrations as a biomarker for assessing long-term activity of the hypothalamic-pituitary-adrenocortical (HPA) axis and chronic stress, will aid in the collection, analysis, and interpretation of the cortisol results (see LOS). Baseline hair samples will be collected by study staff; 6 month samples will be collected by the caregiver and mailed to the study team (detailed instructions will be provided to caregivers for collection). We chose hair (instead of saliva, for instance) primarily due to the ease of data collection, all while being able to accurately collect our construct of interest (e.g., saliva often requires collection multiple times per day over a series of days).

Mindfulness will be measured via two self-report measures. The Mindful Attention Awareness Scale (MAAS) assesses trait mindfulness, specifically the attentional component, on a 6-point Likert scale.140 The Five Facet Mindfulness Questionnaire – Short Form (FFMQ-SF) assesses additional facets of mindfulness not measured in the MAAS on a 5-point Likert scale: Observing, Describing, Acting with Awareness, Nonjudging/Acceptance, and Nonreactivity.141 Importantly, both the MAAS and the FFMQ significantly increased from baseline to end of treatment, suggesting that FOCUS improved mindfulness as expected in the pilot study.

Self-efficacy will be measured via the Caregiver Self-efficacy Scale (CaSES), which measures caregivers’ confidence in their ability to manage the HCT patient on an 4-point Likert scale. Previous research in other populations has shown that MBIs impact self-efficacy,142-144 and we would expect increases in self-efficacy to ultimately impact caregiver health outcomes. The Duke Functional Social Support Questionnaire (FSSQ) will assess social support on a 5-point Likert scale.145Coping will be assessed by three 6-point Likert items regarding a self-assessment of ability to cope and one multiple response item of coping strategies that have been used.

*Daily Diary*. All caregivers will receive a daily diary prompt to answer 10 questions each evening via the study app. These questions will appear throughout the 6 weeks of caregiver treatment and should take no longer than 2 minutes to complete. Affect will be assessed using six items from the PANAS-X (i.e., stressed, overwhelmed, frustrated, drained, guilty, hopeful).146 Because mindfulness is a multi-dimensional construct, three items adapted from existing measures (MAAS, FFMQ, Toronto Mindfulness Scale) will assess the domains of attention, non-judgment, and decentering.140,141,147 Self-efficacy will be assessed via 1 question derived from the CaSES.

*Alcohol and Tobacco use*. At each assessment, all caregivers will answer four items assessing the quantity and frequency of smoking cigarettes and three items from the Audit-C assessing alcohol use.

*COVID-19.* Because recruitment is taking place during an ongoing pandemic that may add to their overall stress, all caregivers and patients will be asked five items related to their experience with COVID (difficulties, financial stress, inter-personal interactions, loneliness, and testing for COVID) in the prior month; these will be asked at each assessment visit.

*Healthy Living.* In order to measure general wellness, self-care, and healthy living, seven items assessing frequency of healthy eating, exercise, relaxation, balancing demands, sleep, and self-care will be asked. These items are adapted from the Mindful Self-Care measure. Items are rated on a 5-point likert scale (Never to Regularly).

Table 4. Measures

**Treatment Feedback from Caregivers.** Satisfaction with the treatment offered will be measured with the 8-item Client Satisfaction Questionnaire (CSQ). Feedback on the number, timing, and content of strategies sent by the app for the HL and FOCUS conditions will be assessed by four multiple choice items. Feedback on the ease/difficulty and convenience of the app will be measured by three Likert scale items. Additional app feedback will be solicited through one open-ended item. Caregivers in the HL and FOCUS conditions will complete an 8-item Likert scale to assess facilitator characteristics (Working Alliance Inventory; e.g., warmth, empathy).

|  |  |  | **SURVEY/TIMEFRAME** | | | | | **APP DATA** | **CHART** |
| --- | --- | --- | --- | --- | --- | --- | --- | --- | --- |
|  | **Assessment** | **Measure** | **Items** | **Baseline** | **EOT** | **Follow-up 1** | **Follow-up 2** | **Baseline - EOT** |  |
| **Caregiver Measures** | Demographics | Standard | 15 | X |  |  |  |  |  |
|  | Mental health Services |  | 1 | X | X | X | X |  |  |
|  | Burden | ZBI^117^ | 12 | X | X | X | X |  |  |
|  | Depression | CESD^120^ | 20 | X | X | X | X |  |  |
|  | Anxiety | GAD-7^122^ | 8 | X | X | X | X |  |  |
|  | Post Traumatic Growth | PTGI^125^ | 21 | X | X | X | X |  |  |
|  | Stress | PSS^131^ | 14 | X | X | X | X |  |  |
|  |  | IES | 15 | X | X | X | X |  |  |
|  |  | Cortisol | - | X |  |  | X |  |  |
|  | Affect | PANAS^136^ | 20 | X | X | X | X | X |  |
|  | Mindfulness | MAAS^140^ | 15 | X | X | X | X | X |  |
|  |  | FFMQ^141^ | 15 | X | X | X | X | X |  |
|  | Self-Efficacy | CASeS | 21 | X | X | X | X | X |  |
|  | Social Support | FSSQ^145^ | 8 | X | X | X | X |  |  |
|  | Healthy Living | MSC | 7 | X | X | X | X |  |  |
|  | Distress | Thermometer | 1 | X | X | X | X |  |  |
|  | COVID | created | 5 | X | X | X | X |  |  |
|  | Smoking | Q/F | 4 | X | X | X | X |  |  |
|  | Alcohol | Audit-C | 3 | X | X | X | X |  |  |
|  | Coping | new | 4 | X | X | X | X |  |  |
|  | Daily Diary |  | 10 |  |  |  |  | X |  |
|  | App Use |  | - |  |  |  |  | X |  |
|  | Treatment Feedback | CSQ | 8 |  | X |  |  |  |  |
|  |  | WAI-SR (Focus & HL) | 12 |  | X |  |  |  |  |
|  |  | Strategies (Focus) | 20 |  | X |  |  |  |  |
|  |  | Strategies (HL) | 12 |  |  |  |  |  |  |
|  |  | App | 5 |  | X |  |  |  |  |
|  |  | Home practice |  |  |  |  |  |  |  |
| **Patient Measures** | Demographics | Standard | 10 | X |  |  |  |  |  |
|  | Clinical Characteristics | Standard | - | X | X | X | X |  | X |
|  | Distress | CESD^120^ | 20 | X | X | X | X |  |  |
|  |  | GAD-7^122^ | 8 | X | X | X | X |  |  |
|  |  | Thermometer | 1 | X | X | X | X |  |  |
|  | Mental Health Services | Standard | 1 | X | X | X | X |  |  |
|  | COVID | created | 5 | X | X | X | X |  |  |
|  | Patient Interview | Created (n=~30) | 20 |  |  |  | X |  |  |
| **Patient**  **Healthcare Utilization** | Readmissions | - | - |  | X | X | X |  | X |
|  | Readmissions length | - | - |  | X | X | X |  | X |
|  | Unexpected clinic visits | - | - |  | X | X | X |  | X |

**Institutional Review Board**

No subject is to be enrolled on this protocol until the Center’s Institution Review Board has approved it.

# Monitoring

Monitoring plan development for this project is commensurate with the risks proposed by the project.

Monitoring will be ongoing by the principal investigator (PI-Dr. Vinci), and the Institutional Review Boards (IRB) of Advarra. Overall, the plan for monitoring includes: 1) Monitoring the progress of the study; 2) Assuring compliance with the requirements for reporting adverse events that may occur during the study; and 3) assuring data accuracy and protocol compliance. For all study protocols, the PI is responsible for the reporting of adverse events to the IRB.

Dr. Vinci will oversee the implementation of the study and daily monitoring. This will include weekly meetings to discuss any issues related to the progression of the project and factors that may affect the outcome, including a review of data quality and security, recruitment, and retention. Adverse events will also be discussed. A brief report will be created and submitted annually for the study record and submitted to the Advarra IRB. For any problems that may arise, Dr. Vinci will consult with the co-investigators to discuss how to best proceed.

# Informed Consent

The investigators and the research staff are responsible for obtaining consent by the participants.

Caregivers and patients will be scheduled for their consenting session to occur in clinic, to coincide with a patient visit. Study staff will consent caregivers and patients separately. Caregiver consent will occur first, and if the caregiver consents, the patient will be invited to complete the informed consent process. Study staff will provide a detailed description of the study, answer questions, obtain informed consent, and finalize eligibility. Participants will be given as much time as needed to ask questions during the consenting process, prior to agreeing to participate in the study. From there, if the patient declines to participate, the caregiver may still participate. We believe this is the ethical approach (vs dropping the caregiver), as the caregiver already made the decision to participate, was informed about the intervention, and scheduled for treatment visits. Once consented, if either member of the dyad drops, the other will be allowed to remain in the study.

# STATISTICAL CONSIDERATIONS

### Data Management and Statistical Considerations

Descriptive statistics will be used to summarize and evaluate the distributions of all study variables. Transformations will be applied, as needed, prior to hypothesis testing. Missing data analyses will assess 1) prospective predictors of missingness that may influence interpretation and generalizability of results from hypothesis testing and 2) whether or not multiple imputation for managing missing data would be beneficial. Paired comparisons will be used to identify group differences, despite randomization, in demographics, clinical, and psycho-social variables that will then be included as covariates when testing hypotheses. Primary analyses for Aims 1 and 2 will use generalized estimating equations (GEE) with an identity link function, linear regression, and compound symmetry for the working correlation matrix. Advantages to using GEE, which fit population-averaged models, are to 1) assess changes in the outcome variable over time, 2) aggregate measures of the outcome variable across time to capture condition differences that may appear at different times for different participants, 3) assess primary hypotheses with targeted comparisons that incorporate data from all participants across multiple assessments, and 4) help effectively manage missing data by study completers under the missing at random assumption (MAR). The Holm method148 will be applied to control for experiment-wise error in the primary analyses, with alpha set at .025 for tests of FOCUS vs UC and set at .05 for FOCUS vs HL. Primary analyses will be performed using SAS version 9.4 (SAS Institute, Cary, NC).

**Power Estimates**: There will be 90 dyads randomized to each condition, with at least 63 per condition (30% dropout) expected at the 2-month assessment. PASS 16 was used to estimate power and effect size based on this sample size. Aim 1 hypothesizes that average caregiver burden will be lower in the FOCUS vs UC condition at 2 months. With alpha=.025, a two-sided test, and group n’s of 63, it is estimated that power

>.80 to detect a standardized difference between means of 0.56, which can be described as slightly greater than a medium effect size. Aim 1 also hypothesizes that average caregiver burden will be lower in the FOCUS vs HL condition at 2 months. Under the same conditions as above, but with alpha set at .05, it is estimated that power >.80 to detect a standardized difference between means of 0.51, a medium effect size. Power estimates for the hypotheses to be tested in Aim 2 (e.g., patient distress) parallel those for Aim 1. It is possible that fewer patients will agree to participate and the minimum effect sizes will be higher than those for Aim 1 (e.g., if group n’s=50, then effect sizes increase to 0.62 [UC] and 0.57 [HL]).

**Specific Aim 1**: Aim 1 is to evaluate the efficacy of FOCUS when compared to UC and HL on caregiver burden. H1a and H1b: Caregivers in FOCUS will report lower levels of burden 2 months post-treatment than caregivers in UC (H1a) and HL (H1b). The primary predictors of the GEE model are condition (e.g., FOCUS vs UC), time (end of treatment [8 weeks] vs 2 months), and their interaction. The GEE will include as a covariate any baseline measure that differs by group (p<.05) as a potential confound (e.g., age, relationship to patient, baseline burden). Other covariates will be predictors of missing follow-up surveys and/or self-reported burden at 8 weeks or 2 months (p<.05). These covariates will increase the viability of the Missing at Random assumption and improve parameter estimates within a full information maximum likelihood model. As described above, H1a will be assessed at alpha=.025 and H1b at .05. Each hypothesis will be tested using a planned contrast of condition at 2 months assessing the prediction that FOCUS will have a lower mean burden than the comparison condition (UC or HL). A significant main effect for condition would be another indicator of group differences (burden averaged over the 2 assessments).

Additional analyses will be performed to assess the effect of FOCUS on burden at the 6 month follow- up. A new GEE model will incorporate the three assessments of burden (8 weeks, 2 months, and 6 months) using appropriate covariates. The primary test will be a planned contrast of condition at 6 months. A separate model is justified by 1) the expected increase in missing surveys at 6 months and 2) the expected addition of covariates that predict missing surveys and/or burden at 6 months. This test at the 6-month follow-up will also be conducted for aim 2 outcomes of patient distress and healthcare utilization.

**Specific Aim 2**: Aim 2 is to evaluate the impact of FOCUS on patient health and patient healthcare utilization. H2a and H2b: Patients of caregivers in FOCUS will report lower levels of distress 2 months post- treatment than patients of caregivers in UC (H2a) or HL (H2b). Exploratory: Determine the impact of FOCUS on patient healthcare utilization in the first 2 months and from 3 to 6 months post-treatment (hospital readmissions, length of readmissions, unexpected patient clinic visits). The analysis for this hypothesis parallels that of Aim 1. The main differences are the outcome measure and the sample. If preliminary analyses show that distress exhibits sufficient characteristics to be treated as a continuous variable, then the identity link and linear regression will be used within the GEE. If not, an appropriate link function and regression approach will be applied. The sample of patients has potential to have more missing surveys, to have different predictors of missing surveys, and to have unique predictors of distress. Therefore, these GEE models are likely to have different sets of covariates (e.g., number of serious transplant complications; acute GVHD; relapse).

Similar GEE models will be used to explore group differences in patient healthcare utilization through 2 months and from 3 to 6 months. As noted above, model covariates may differ. The main difference in these models will be driven by the distribution characteristics of the healthcare utilization measures. For those measures that cannot be considered continuous, the link function and regression type within the GEE will be changed to an option that best matches the outcome distribution (e.g., link=binary, regression=logistic for whether or not there were unexpected patient clinic visits).

**Specific Aim 3**: Aim 3 is to evaluate putative mechanisms and moderators of FOCUS. Prospective mechanisms of lower perceived burden are higher mindfulness and self-efficacy, and lower stress and negative affect. The measure of these variables at 2 months (6 months for cortisol) post-treatment will be evaluated using the same approach described for the analyses in Aim 1.

These variables will also be assessed within the daily diary component of the study, which allows for 2 additional opportunities to assess mechanisms. First, the repeated, daily measures will be assessed using GEE for condition comparison with an emphasis on the main effect of condition (averaged over the multiple daily measures) and the condition x time interaction (diverging trajectories with the group differences increasing over time). Second, the repeated, daily measures of an active mechanism variable and the measures of burden at 8 weeks and 2 months will be entered into a structural equation model designed to evaluate whether or not changes in an active mechanism variable precede changes in burden. Mplus version

8.4 will be used for these models.149

Potential moderators (e.g., gender, age, relationship to patient, income, proximity to cancer center [defined as the mileage of home residence from Moffitt], baseline caregiver distress) of FOCUS on caregiver burden will be evaluated by adding to the GEE model for Aim 1 the moderator (e.g., gender) and its interaction with condition. A significant interaction will be further explored by testing differences in burden for each condition within appropriate subsets of the moderator (e.g., male and female).

**Data management**. Study data will be managed through REDCap (Research Electronic Data Capture), a data management software system supported and provided free by Moffitt. REDCap allows creation of customized, secure data management systems including web-based data entry forms, reporting tools, and a full array of security features including user and group-based privileges with a full audit trail of data manipulation and export procedures. Nationally, REDCap software is developed, enhanced, and supported through a multi- institutional consortium led by Vanderbilt University. The Project Manager, along with the Research Coordinator, will oversee creation of the database, questionnaires, and an automated messaging system.

### Weekly reports will be generated for review to assure timely completion of study goals. REDCap will be used to implement randomization.

### REFERENCES

1. Gyurkocza B, Rezvani A, Storb RF. Allogeneic hematopoietic cell transplantation: the state of the art.

*Expert Rev Hematol.* 2010;3(3):285-299.

1. Gemmill R, Cooke L, Williams AC, Grant M. Informal caregivers of hematopoietic cell transplant patients: A review and recommendations for interventions and research. *Cancer Nurs.* 2011;34(6):E13.
2. Wulff-Burchfield E, Jagasia M, Savani B. Long-term follow-up of informal caregivers after allo-SCT: A systematic review. *Bone Marrow Transplant.* 2013;48(4):469.
3. Bishop MM, Beaumont JL, Hahn EA, et al. Late effects of cancer and hematopoietic stem-cell transplantation on spouses or partners compared with survivors and survivor-matched controls. *J Clin Oncol.* 2007;25(11):1403-1411.
4. Jim HS, Quinn GP, Gwede CK, et al. Patient education in allogeneic hematopoietic cell transplant: What patients wish they had known about quality of life. *Bone Marrow Transplant.* 2014;49(2):299-303.
5. Simoneau TL, Mikulich‐Gilbertson SK, Natvig C, et al. Elevated peri‐transplant distress in caregivers of allogeneic blood or marrow transplant patients. *Psycho‐Oncology.* 2013;22(9):2064-2070.
6. Ankuda CK, Maust DT, Kabeto MU, McCammon RJ, Langa KM, Levine DA. Association Between Spousal Caregiver Well‐Being and Care Recipient Healthcare Expenditures. *J Am Geriatr Soc.* 2017;65(10):2220-2226.
7. Dionne‐Odom JN, Hull JG, Martin MY, et al. Associations between advanced cancer patients' survival and family caregiver presence and burden. *Cancer Medicine.* 2016;5(5):853-862.
8. Hochhausen N, Altmaier EM, McQuellon R, et al. Social support, optimism, and self-efficacy predict physical and emotional well-being after bone marrow transplantation. *J Psychosoc Oncol.* 2007;25(1):87-101.
9. Jacobs JM, Shaffer KM, Nipp RD, et al. Distress is interdependent in patients and caregivers with newly diagnosed incurable cancers. *Ann Behav Med.* 2017;51(4):519-531.
10. Rini C, Redd WH, Austin J, et al. Effectiveness of partner social support predicts enduring psychological distress after hematopoietic stem cell transplantation. *J Consult Clin Psychol.* 2011;79(1):64.
11. Brown SL, Smith DM, Schulz R, et al. Caregiving behavior is associated with decreased mortality risk.

*Psychol Sci.* 2009;20(4):488-494.

## Kim Y, Schulz R, Carver CS. Benefit finding in the cancer caregiving experience. *Psychosom Med.*

2007;69(3):283-291.

1. Li Q, Loke AY. The positive aspects of caregiving for cancer patients: a critical review of the literature and directions for future research. *Psycho‐Oncology.* 2013;22(11):2399-2407.
2. Ownsworth T, Henderson L, Chambers SK. Social support buffers the impact of functional impairments on caregiver psychological well‐being in the context of brain tumor and other cancers. *Psycho‐ Oncology.* 2010;19(10):1116-1122.
3. Northouse L, Williams A-l, Given B, McCorkle R. Psychosocial care for family caregivers of patients with cancer. *J Clin Oncol.* 2012;30(11):1227-1234.
4. Breslin FC, Zack M, McMain S. An information-processing analysis of mindfulness: Implications for relapse prevention in the treatment of substance abuse. *Clinical Psychology: Science and Practice.* 2002;9:275-299.

## Carmody J, Baer RA, LB Lykins E, Olendzki N. An empirical study of the mechanisms of mindfulness in a mindfulness‐based stress reduction program. *J Clin Psychol.* 2009;65(6):613-626.

1. Creswell JD, Lindsay EK. How does mindfulness training affect health? A mindfulness stress buffering account. *Curr Dir Psychol Sci.* 2014;23(6):401-407.
2. Grabovac AD, Lau MA, Willett BR. Mechanisms of mindfulness: A Buddhist psychological model.

*Mindfulness.* 2011;2(3):154-166.

## Hölzel BK, Lazar SW, Gard T, Schuman-Olivier Z, Vago DR, Ott U. How does mindfulness meditation work? Proposing mechanisms of action from a conceptual and neural perspective. *Perspect Psychol Sci.* 2011;6(6):537-559.

1. Tang Y-Y, Hölzel BK, Posner MI. The neuroscience of mindfulness meditation. *Nature Reviews Neuroscience.* 2015;16(4):213-225.

## Laudenslager ML, Simoneau TL, Kilbourn K, et al. A randomized control trial of a psychosocial intervention for caregivers of allogeneic hematopoietic stem cell transplant patients: effects on distress. *Bone Marrow Transplant.* 2015;50(8):1110.

1. O'toole MS, Zachariae R, Renna ME, Mennin DS, Applebaum A. Cognitive behavioral therapies for informal caregivers of patients with cancer and cancer survivors: a systematic review and meta‐analysis. *Psycho‐oncology.* 2017;26(4):428-437.
2. El‐Jawahri A, Jacobs JM, Nelson AM, et al. Multimodal psychosocial intervention for family caregivers of patients undergoing hematopoietic stem cell transplantation: A randomized clinical trial. *Cancer.* 2020.
3. Arch JJ, Craske MG. Mechanisms of mindfulness: Emotion regulation following a focused breathing induction. *Behavior Research and Therapy.* 2006;44(12):1849-1858.
4. Davis JM, Fleming MF, Bonus KA, Baker TB. A pilot study on mindfulness based stress reduction for smokers. *BMC Complement Altern Med.* 2007;7:2.
5. Gotink RA, Chu P, Busschbach JJ, Benson H, Fricchione GL, Hunink MM. Standardised mindfulness- based interventions in healthcare: An overview of systematic reviews and meta-analyses of RCTs. *PLoS One.* 2015;10(4):e0124344.
6. Ortner CNM, Kilner SJ, Zelazo PD. Mindfulness meditation and reduced emotional interference on a cognitive task. *Motivation and Emotion.* 2007;31(4):271-283.
7. Tang YY, Ma Y, Wang J, et al. Short-term meditation training improves attention and self-regulation.

*Proc Natl Acad Sci U S A.* 2007;104(43):17152-17156.

## Vinci C, Peltier MR, Shah S, et al. Effects of a brief mindfulness intervention on negative affect and urge to drink among college student drinkers. *Behavior Research and Therapy.* 2014;59:82-93.

1. Matousek RH, Dobkin PL, Pruessner J. Cortisol as a marker for improvement in mindfulness-based stress reduction. *Complement Ther Clin Pract.* 2010;16(1):13-19.
2. Sanada K, Montero-Marin J, Díez MA, et al. Effects of Mindfulness-based interventions on salivary cortisol in healthy adults: A meta-analytical review. *Front Physiol.* 2016;7.
3. Birnie K, Garland SN, Carlson LE. Psychological benefits for cancer patients and their partners participating in mindfulness‐based stress reduction (MBSR). *Psycho‐Oncology.* 2010;19(9):1004-1009.
4. Fish JA, Ettridge K, Sharplin G, Hancock B, Knott V. M indfulness‐based C ancer S tress M anagement: impact of a mindfulness‐based programme on psychological distress and quality of life. *European journal of cancer care.* 2014;23(3):413-421.
5. van den Hurk DG, Schellekens MP, Molema J, Speckens AE, van der Drift MA. Mindfulness-Based Stress Reduction for lung cancer patients and their partners: Results of a mixed methods pilot study. *Palliat Med.* 2015;29(7):652-660.
6. Kubo A, Altschuler A, Kurtovich E, et al. A pilot mobile-based mindfulness intervention for cancer patients and their informal caregivers. *Mindfulness.* 2018;9(6):1885-1894.
7. Lengacher CA, Kip KE, Barta M, et al. A pilot study evaluating the effect of mindfulness-based stress reduction on psychological status, physical status, salivary cortisol, and interleukin-6 among advanced- stage cancer patients and their caregivers. *J Holist Nurs.* 2012;30(3):170-185.
8. Vinci C, Pidala J, Lau P, Reblin M, Jim H. A mindfulness-based intervention for caregivers of allogeneic hematopoietic stem cell transplant patients: Pilot results *Psychooncology.* accepted with minor revisions.
9. Kent EE, Rowland JH, Northouse L, et al. Caring for caregivers and patients: research and clinical priorities for informal cancer caregiving. *Cancer.* 2016;122(13):1987-1995.
10. Fletcher BS, Miaskowski C, Given B, Schumacher K. The cancer family caregiving experience: an updated and expanded conceptual model. *Eur J Oncol Nurs.* 2012;16(4):387-398.
11. Manne S, Ostroff J, Winkel G, Goldstein L, Fox K, Grana G. Posttraumatic growth after breast cancer: Patient, partner, and couple perspectives. *Psychosom Med.* 2004;66(3):442-454.
12. Widows MR, Jacobsen PB, Booth-Jones M, Fields KK. Predictors of posttraumatic growth following bone marrow transplantation for cancer. *Health Psychol.* 2005;24(3):266.
13. Vinci C, Reblin M, Bulls H, et al. Understanding coping strategies of cancer caregivers to inform mindfulness-based interventions: A qualitative study. *European Journal of Integrative Medicine.* 2019;30:100936.

## Simoneau TL, Kilbourn K, Spradley J, Laudenslager ML. An evidence-based stress management intervention for allogeneic hematopoietic stem cell transplant caregivers: Development, feasibility and acceptability. *Support Care Cancer.* 2017.

1. Bevans M, Wehrlen L, Castro K, et al. A problem-solving education intervention in caregivers and patients during allogeneic hematopoietic stem cell transplantation. *J Health Psychol.* 2014;19(5):602- 617.
2. Kabat-Zinn J. *Full catastrophe living: Using the wisdom of your body and mind to face stress, pain, and illness.* Random House LLC; 1990.
3. Kabat-Zinn J. *Wherever you go, there you are: Mindfulness in everyday life.* New York, NY: Hyperion; 1994.

## Teasdale JD, Segal Z, Williams JMG. How does cognitive therapy prevent depressive relapse and why should attentional control (mindfulness) training help? *Behav Res Ther.* 1995;33(1):25-39.

1. Wood AW, Gonzalez J, Barden SM. Mindful caring: using mindfulness-based cognitive therapy with caregivers of cancer survivors. *J Psychosoc Oncol.* 2015;33(1):66-84.
2. Brown KW, Weinstein N, Creswell JD. Trait mindfulness modulates neuroendocrine and affective responses to social evaluative threat. *Psychoneuroendocrinology.* 2012;37(12):2037-2041.
3. Garland E, Gaylord S, Park J. The role of mindfulness in positive reappraisal. *Explore: The Journal of Science and Healing.* 2009;5(1):37-44.

## Garland EL, Gaylord SA, Fredrickson BL. Positive reappraisal mediates the stress-reductive effects of mindfulness: An upward spiral process. *Mindfulness.* 2011;2(1):59-67.

1. Garland EL, Geschwind N, Peeters F, Wichers M. Mindfulness training promotes upward spirals of positive affect and cognition: Multilevel and autoregressive latent trajectory modeling analyses. *Front Psychol.* 2015;6:15.
2. Bejanyan N, Bolwell BJ, Lazaryan A, et al. Risk factors for 30-day hospital readmission following myeloablative allogeneic hematopoietic cell transplantation (allo-HCT). *Biol Blood Marrow Transplant.* 2012;18(6):874-880.
3. Rauenzahn S, Truong Q, Cumpston A, et al. Predictors and impact of thirty-day readmission on patient outcomes and health care costs after reduced-toxicity conditioning allogeneic hematopoietic cell transplantation. *Biol Blood Marrow Transplant.* 2014;20(3):415-420.
4. Spring L, Li S, Soiffer RJ, Antin JH, Alyea III EP, Glotzbecker B. Risk factors for readmission after allogeneic hematopoietic stem cell transplantation and impact on overall survival. *Biol Blood Marrow Transplant.* 2015;21(3):509-516.
5. Grant M, Cooke L, Bhatia S, Forman SJ. Discharge and unscheduled readmissions of adult patients undergoing hematopoietic stem cell transplantation: implications for developing nursing interventions. Paper presented at: Oncology nursing forum2005.
6. Richardson DR, Huang Y, McGinty HL, et al. Psychosocial risk predicts high readmission rates for hematopoietic cell transplant recipients. *Bone Marrow Transplant.* 2018;53(11):1418.
7. El‐Jawahri A, Chen YB, Brazauskas R, et al. Impact of pre‐transplant depression on outcomes of allogeneic and autologous hematopoietic stem cell transplantation. *Cancer.* 2017;123(10):1828-1838.
8. Aycock DM, Hayat MJ, Helvig A, Dunbar SB, Clark PC. Essential considerations in developing attention control groups in behavioral research. *Res Nurs Health.* 2018;41(3):320-328.
9. Goldberg SB, Manley AR, Smith SS, et al. Hair cortisol as a biomarker of stress in mindfulness training for smokers. *The Journal of Alternative and Complementary Medicine.* 2014;20(8):630-634.

## Vinci C, Reblin M, Jim H, Pidala J, Bulls H, Cutolo E. Understanding preferences for a mindfulness- based stress management program among caregivers of hematopoietic cell transplant patients. *Complement Ther Clin Pract.* 2018;33:164-169. PMCID: PMC6435265.

1. Vinci C, Copeland AL, Carrigan MH. Exposure to negative affect cues and urge to smoke. *Exp Clin Psychopharmacol.* 2012;20(1):47-55.
2. Vinci C, Guo L, Spears CA, et al. Socioeconomic indicators as predictors of smoking cessation among Spanish-speaking Mexican Americans. *Ethn Health.* 2017.
3. Vinci C, Spears CA, Peltier MR, Copeland AL. Facets of mindfulness mediate the relationship between depressive symptoms and smoking behavior. *Mindfulness.* 2016;7(6):1408-1415. **PMCID: PMC5222556**
4. Cambron C, Haslam AK, Baucom BR, et al. Momentary precipitants connecting stress and smoking lapse during a quit attempt. *Health Psychol.* 2019.
5. Vinci C, Haslam A, Lam CY, Kumar S, Wetter DW. The use of ambulatory assessment in smoking cessation. *Addict Behav.* 2018;83:18-24. PMCID: PMC5964000
6. Vinci C, Li L, Guo L, et al. The association of positive emotion and first smoking lapse: An ecological momentary assessment study. *Health Psychol.* 2017. **PMCID: PMC5653435**
7. Reblin M, Wu YP, Pok J, et al. Development of the electronic social network assessment program using the center for eHealth and wellbeing research roadmap. *JMIR human factors.* 2017;4(3):e23.
8. Terrill AL, MacKenzie JJ, Reblin M, Einerson J, Ferraro J, Altizer R. A Collaboration Between Game Developers and Rehabilitation Researchers to Develop a Web-Based App for Persons With Physical Disabilities: Case Study. *JMIR Rehabilitation and Assistive Technologies.* 2019;6(2):e13511.
9. Otto AK, Ketcher D, Heyman RE, Vadaparampil ST, Ellington L, Reblin M. Communication between Advanced Cancer Patients and Their Family Caregivers: Relationship with Caregiver Burden and Preparedness for Caregiving. *Health Communication.* 2020:1-8.
10. Reblin M, Ketcher D, Forsyth P, et al. Outcomes of an electronic social network intervention with neuro-oncology patient family caregivers. *J Neurooncol.* 2018;139(3):643-649.
11. Reblin M, Small B, Jim H, Weimer J, Sherwood P. Mediating burden and stress over time: Caregivers of patients with primary brain tumor. *Psycho‐Oncology.* 2018;27(2):607-612.
12. Reblin M, Sutton SK, Vadaparampil ST, Heyman RE, Ellington L. Behind closed doors: How advanced cancer couples communicate at home. *J Psychosoc Oncol.* 2019;37(2):228-241.
13. Hoogland AI, Bulls HW, Gonzalez BD, et al. Circadian Rhythmicity as a Predictor of Quality of Life in Allogeneic Hematopoietic Cell Transplant Patients. *J Pain Symptom Manage.* 2019;57(5):952-960. e951.
14. Jim HS, Small B, Hartman S, et al. Clinical predictors of cognitive function in adults treated with hematopoietic cell transplantation. *Cancer.* 2012;118(13):3407-3416.
15. Jim HS, Sutton S, Majhail NS, et al. Severity, course, and predictors of sleep disruption following hematopoietic cell transplantation: a secondary data analysis from the BMT CTN 0902 trial. *Bone Marrow Transplant.* 2018;53(8):1038.
16. Liang J, Lee SJ, Storer BE, et al. Rates and Risk Factors for Post-Traumatic Stress Disorder Symptomatology among Adult Hematopoietic Cell Transplant Recipients and Their Informal Caregivers. *Biol Blood Marrow Transplant.* 2019;25(1):145-150.
17. Nelson AM, Jim HS, Small BJ, et al. Sleep disruption among cancer patients following autologous hematopoietic cell transplantation. *Bone Marrow Transplant.* 2018;53(3):307-314.
18. Phillips KM, McGinty HL, Cessna J, et al. A systematic review and meta-analysis of changes in cognitive functioning in adults undergoing hematopoietic cell transplantation. *Bone Marrow Transplant.* 2013;48(10):1350.
19. Wood WA, Le‐Rademacher J, Syrjala KL, et al. Patient‐reported physical functioning predicts the success of hematopoietic cell transplantation (BMT CTN 0902). *Cancer.* 2016;122(1):91-98.
20. Pidala J, Anasetti C, Jim H. Quality of life after allogeneic hematopoietic cell transplantation. *Blood, The Journal of the American Society of Hematology.* 2009;114(1):7-19.

## Pidala J, Kim J, Alsina M, et al. Prolonged sirolimus administration after allogeneic hematopoietic cell transplantation is associated with decreased risk for moderate-severe chronic graft-versus-host disease. *Haematologica.* 2015;100(7):970-977.

1. Pidala J, Kim J, Jim H, et al. A randomized phase II study to evaluate tacrolimus in combination with sirolimus or methotrexate after allogeneic hematopoietic cell transplantation. *Haematologica.* 2012;97(12):1882-1889.
2. Pidala J, Kurland B, Chai X, et al. Patient-reported quality of life is associated with severity of chronic graft-versus-host disease as measured by NIH criteria: report on baseline data from the Chronic GVHD Consortium. *Blood, The Journal of the American Society of Hematology.* 2011;117(17):4651-4657.
3. Pidala J, Lee SJ, Quinn G, Jim H, Kim J, Anasetti C. Variation in management of immune suppression after allogeneic hematopoietic cell transplantation. *Biol Blood Marrow Transplant.* 2011;17(10):1528- 1536.
4. Pidala J, Martens M, Anasetti C, et al. Factors Associated With Successful Discontinuation of Immune Suppression After Allogeneic Hematopoietic Cell Transplantation. *JAMA oncology.* 2020;6(1):e192974- e192974.
5. Decook L, Chang Y, Slack J, et al. Association of hematopoietic cell transplantation-specific comorbidity index with resource utilization after allogeneic transplantation. *Bone Marrow Transplant.* 2017;52(7):998-1002.
6. Khera N, Albelda R, Hahn T, et al. Financial Hardship after Hematopoietic Cell Transplantation: Lack of Impact on Survival. *Cancer Epidemiology and Prevention Biomarkers.* 2018;27(3):345-347.

## Khera N, Chang Y-h, Hashmi S, et al. Financial burden in recipients of allogeneic hematopoietic cell transplantation. *Biol Blood Marrow Transplant.* 2014;20(9):1375-1381.

1. Khera N, Chang Y-H, Slack J, et al. Impact of race and ethnicity on outcomes and health care utilization after allogeneic hematopoietic cell transplantation. *Leuk Lymphoma.* 2015;56(4):987-992.
2. Khera N, Chow EJ, Leisenring WM, et al. Factors associated with adherence to preventive care practices among hematopoietic cell transplantation survivors. *Biol Blood Marrow Transplant.* 2011;17(7):995- 1003.
3. Khera N, Martin P, Edsall K, et al. Patient-centered care coordination in hematopoietic cell transplantation. *Blood advances.* 2017;1(19):1617-1627.
4. Khera N, Storer B, Flowers ME, et al. Nonmalignant late effects and compromised functional status in survivors of hematopoietic cell transplantation. *J Clin Oncol.* 2012;30(1):71.
5. Khera N, Storer B, Sandmaier BM, Chapko MK, Lee SJ. Costs of second allogeneic hematopoietic cell transplantation. *Transplantation.* 2013;96(1):108.
6. Brandon TH, Simmons VN, Meade CD, et al. Self-help booklets for preventing postpartum smoking relapse: A randomized trial. *Am J Public Health.* 2012;102(11):2109-2115.
7. Brandon TH, Simmons VN, Sutton SK, et al. Extended self-help for smoking cessation: A randomized controlled trial. *Am J Prev Med.* 2016;51(1):54-62.
8. Gwede CK, Sutton SK, Chavarria EA, et al. A culturally and linguistically salient pilot intervention to promote colorectal cancer screening among Latinos receiving care in a Federally Qualified Health Center. *Health Educ Res.* 2019;34(3):310-320.
9. Medina-Ramírez P, Sutton SK, Martínez Ú, et al. A randomized controlled trial of a smoking cessation self-help intervention for Spanish-speaking Hispanic/Latinx smokers: Study design and baseline characteristics. *Contemp Clin Trials.* 2019;85:105836.
10. Meltzer LR, Simmons VN, Sutton SK, et al. A randomized controlled trial of a smoking cessation self- help intervention for dual users of tobacco cigarettes and e-cigarettes: Intervention development and research design. *Contemp Clin Trials.* 2017;60:56-62.
11. Simmons VN, Sutton SK, Quinn GP, Meade CD, Brandon TH. Prepartum and postpartum predictors of smoking. *Nicotine & Tobacco Research.* 2014;16(4):461-468.
12. Teasdale JD, Segal ZV, Williams JMG, Ridgeway VA, Soulsby JM, Lau MA. Prevention of relapse/recurrence in major depression by mindfulness-based cognitive therapy. *J Consult Clin Psychol.* 2000;68(4):615-623.
13. Cimprich B, Janz NK, Northouse L, Wren PA, Given B, Given CW. Taking CHARGE: A self‐ management program for women following breast cancer treatment. *Psycho‐Oncology.* 2005;14(9):704- 717.
14. Cooke L, Grant M, Eldredge DH, Maziarz RT, Nail LM. Informal caregiving in hematopoietic blood and marrow transplant patients. *Eur J Oncol Nurs.* 2011;15(5):500-507.
15. Eldredge LKB, Markham CM, Ruiter RA, Kok G, Parcel GS. *Planning health promotion programs: an intervention mapping approach.* San Fransisco, CA: John Wiley & Sons; 2016.
16. Champlin R. Selection of autologous or allogeneic transplantation. *Holland-Frei cancer medicine, 6th edn BC Decker, Hamilton (ON).* 2003.

## Ellington L, Reblin M, Clayton MF, Berry P, Mooney K. Hospice nurse communication with patients with cancer and their family caregivers. *J Palliat Med.* 2012;15(3):262-268.

1. Reblin M, Clayton MF, John KK, Ellington L. Addressing methodological challenges in large communication data sets: Collecting and coding longitudinal interactions in home hospice cancer care. *Health communication.* 2016;31(7):789-797.
2. Reblin M, Heyman RE, Ellington L, Baucom BR, Georgiou PG, Vadaparampil ST. Everyday couples’ communication research: Overcoming methodological barriers with technology. *Patient Educ Couns.* 2018;101(3):551-556.
3. Ransom S, Azzarello LM, McMillan SC. Methodological issues in the recruitment of cancer pain patients and their caregivers. *Research in Nursing & Health.* 2006;29(3):190-198.
4. Steinhauser KE, Clipp EC, Hays JC, et al. Identifying, recruiting, and retaining seriously-ill patients and their caregivers in longitudinal research. *Palliat Med.* 2006;20(8):745-754.
5. Northouse LL, Rosset T, Phillips L, Mood D, Schafenacker A, Kershaw T. Research with families facing cancer: the challenges of accrual and retention. *Res Nurs Health.* 2006;29(3):199-211.
6. Ketcher D, Trettevik R, Vadaparampil ST, Heyman RE, Ellington L, Reblin M. Caring for a spouse with advanced cancer: similarities and differences for male and female caregivers. *J Behav Med.* 2019:1-12.
7. American Cancer Society. Caregiver Resource Guide: Caring for a Loved One with Cancer. 2019; [https://www.cancer.org/content/dam/cancer-org/cancer-control/en/booklets-flyers/american-cancer-](http://www.cancer.org/content/dam/cancer-org/cancer-control/en/booklets-flyers/american-cancer-) society-caregiver-resource-guide.pdf.
8. Chawla N, Collins S, Bowen S, et al. The mindfulness-based relapse prevention adherence and competence scale: Development, interrater reliability, and validity. *Psychotherapy Research.* 2010;20(4):388-397.
9. Bédard M, Molloy DW, Squire L, Dubois S, Lever JA, O'Donnell M. The Zarit Burden Interview: a new short version and screening version. *The gerontologist.* 2001;41(5):652-657.
10. Higginson IJ, Gao W, Jackson D, Murray J, Harding R. Short-form Zarit Caregiver Burden Interviews were valid in advanced conditions. *J Clin Epidemiol.* 2010;63(5):535-542.
11. Gaugler JE, Mittelman MS, Hepburn K, Newcomer R. Clinically significant changes in burden and depression among dementia caregivers following nursing home admission. *BMC Med.* 2010;8(1):85.
12. Radloff LS. The CES-D scale: A self-report depression scale for research in the general population. *Appl Psychol Meas.* 1977;1(3):385-401.
13. Hagedoorn M, Sanderman R, Bolks HN, Tuinstra J, Coyne JC. Distress in couples coping with cancer: A meta-analysis and critical review of role and gender effects. *Psychol Bull.* 2008;134(1):1.
14. Spitzer RL, Kroenke K, Williams JB, Löwe B. A brief measure for assessing generalized anxiety disorder: The GAD-7. *Arch Intern Med.* 2006;166(10):1092-1097.
15. Oechsle K, Goerth K, Bokemeyer C, Mehnert A. Anxiety and depression in caregivers of terminally ill cancer patients: impact on their perspective of the patients' symptom burden. *J Palliat Med.* 2013;16(9):1095-1101.
16. Ullrich A, Ascherfeld L, Marx G, Bokemeyer C, Bergelt C, Oechsle K. Quality of life, psychological burden, needs, and satisfaction during specialized inpatient palliative care in family caregivers of advanced cancer patients. *BMC Palliat Care.* 2017;16(1):31.
17. Tedeschi RG, Calhoun LG. The Posttraumatic Growth Inventory: Measuring the positive legacy of trauma. *J Trauma Stress.* 1996;9(3):455-471.
18. Barata A, Gonzalez BD, Sutton SK, et al. Coping strategies modify risk of depression associated with hematopoietic cell transplant symptomatology. *J Health Psychol.* 2018;23(8):1028-1037.
19. Hann D, Winter K, Jacobsen P. Measurement of depressive symptoms in cancer patients: evaluation of the Center for Epidemiological Studies Depression Scale (CES-D). *J Psychosom Res.* 1999;46(5):437- 443.
20. Piet J, Würtzen H, Zachariae R. The effect of mindfulness-based therapy on symptoms of anxiety and depression in adult cancer patients and survivors: A systematic review and meta-analysis. *J Consult Clin Psychol.* 2012;80(6):1007.
21. Johns SA, Brown LF, Beck-Coon K, et al. Randomized controlled pilot trial of mindfulness-based stress reduction compared to psychoeducational support for persistently fatigued breast and colorectal cancer survivors. *Support Care Cancer.* 2016;24(10):4085-4096.
22. Mosher CE, Winger JG, Hanna N, et al. Randomized pilot trial of a telephone symptom management intervention for symptomatic lung cancer patients and their family caregivers. *J Pain Symptom Manage.* 2016;52(4):469-482.
23. Cohen S, Kamarck T, Mermelstein R. A global measure of perceived stress. *J Health Soc Behav.*

## 1983:385-396.

1. Barakat LP, Alderfer MA, Kazak AE. Posttraumatic growth in adolescent survivors of cancer and their mothers and fathers. *J Pediatr Psychol.* 2005;31(4):413-419.
2. Creamer M, Bell R, Failla S. Psychometric properties of the Impact of Event Scale—Revised. *Behav Res Ther.* 2003;41(12):1489-1496.
3. Goldzweig G, Hubert A, Walach N, et al. Gender and psychological distress among middle-and older- aged colorectal cancer patients and their spouses: An unexpected outcome. *Crit Rev Oncol Hematol.* 2009;70(1):71-82.
4. Weiss DS. The Impact of Event Scale: Revised. *Cross-Cultural Assessment of Psychological Trauma and PTSD*: Springer; 2007:219-238.

## Watson D, Clark LA, Tellegen A. Development and validation of brief measures of positive and negative affect: The PANAS scales. *J Pers Soc Psychol.* 1988;54(6):1063-1070.

1. Meyer J, Novak M, Hamel A, Rosenberg K. Extraction and analysis of cortisol from human and monkey hair. *JoVE (Journal of Visualized Experiments).* 2014(83):e50882.

## Meyer JS, Novak MA. Minireview: hair cortisol: a novel biomarker of hypothalamic-pituitary- adrenocortical activity. *Endocrinology.* 2012;153(9):4120-4127.

1. Sauvé B, Koren G, Walsh G, Tokmakejian S, Van Uum SH. Measurement of cortisol in human hair as a biomarker of systemic exposure. *Clinical and Investigative Medicine.* 2007:E183-E191.

## Brown KW, Ryan RM. The benefits of being present: Mindfulness and its role in psychological well- being. *J Pers Soc Psychol.* 2003;84(4):822-848.

1. Baer RA, Smith GT, Hopkins J, Krietemeyer J, Toney L. Using self-report assessment methods to explore facets of mindfulness. *Assessment.* 2006;13(1):27-45.
2. Britton WB, Bootzin RR, Cousins JC, Hasler BP, Peck T, Shapiro SL. The contribution of mindfulness practice to a multicomponent behavioral sleep intervention following substance abuse treatment in adolescents: A treatment-development study. *Subst Abus.* 2010;31(2):86-97.
3. Chang VY, Palesh O, Caldwell R, et al. The effects of a mindfulness-based stress reduction program on stress, mindfulness self-efficacy, and positive states of mind. *Stress and Health.* 2004;20(3):141-147.
4. Spears C, Hedeker D, Li L, et al. Mechanisms underlying mindfulness-based addiction treatment versus cognitive behavioral therapy and usual care for smoking cessation. *J Consult Clin Psychol.* 2017;85:1029-1040.
5. Broadhead W, Gehlbach SH, De Gruy FV, Kaplan BH. The Duke-UNC Functional Social Support Questionnaire: Measurement of social support in family medicine patients. *Med Care.* 1988:709-723.
6. Watson D, Clark LA. The PANAS-X: Manual for the positive and negative affect schedule-expanded form. 1999.
7. Lau MA, Bishop SR, Segal ZV, et al. The toronto mindfulness scale: Development and validation. *J Clin Psychol.* 2006;62(12):1445-1467.
8. Holm S. A simple sequentially rejective multiple test procedure. *Scandinavian journal of statistics.*

## 1979:65-70.

1. Muthén LK, Muthén BO. 1998–2010 Mplus user‘s guide. *Muthén and Muthén.* 2010.
