# Supplementary material for: Correction: A mindfulness-based stress management program for caregivers of allogeneic hematopoietic stem cell transplant (HCT) patients: Protocol for a randomized controlled trial
Source: PLoS One. 2022 Sep 23;17(9):e0275398. doi: 10.1371/journal.pone.0275398 (PMC9506639; doi:10.1371/journal.pone.0275398)
Supplement: S1 File — (PDF) [file pone.0275398.s001.pdf]

PLOS ONE

STUDY PROTOCOL

A mindfulness-based stress management program for caregivers of allogeneic hematopoietic stem cell transplant (HCT) patients: Protocol for a randomized controlled trial

Min-Jeong Yang[1], Valerie V. Yepez[1], Karen O. Brandon[1,2], Maija Reblin[3], Joseph Pidala[4], Heather S. L. Jim[1], Jerrold S. Meyer[5], L. Robert Gore[1,6], Nandita Khera[7,8], Penny Lau[9], Rachel M. Sauls[1], Sarah R. Jones[1], Christine Vinci[1,2]*

1 Department of Health Outcomes and Behavior, Moffitt Cancer Center, Tampa, Florida, United States of America, **2** Department of Psychology, University of South Florida, Tampa, Florida, United States of America, **3** College of Medicine, University of Vermont, Burlington, Vermont, United States of America, **4** Department of Blood & Marrow Transplantation, Moffitt Cancer Center, Tampa, Florida, United States of America, **5** Department of Psychological & Brain Sciences, University of Massachusetts, Amherst, Massachusetts, United States of America, **6** Department of Biostatistics and Bioinformatics, Moffitt Cancer Center, Tampa, Florida, United States of America, **7** Division of Hematology and Oncology, Mayo Clinic, Phoenix, Arizona, United States of America, **8** College of Medicine, Mayo Clinic, Phoenix, Arizona, United States of America, **9** Department of Social Work, Moffitt Cancer Center, Tampa, Florida, United States of America

* Christine.Vinci@moffitt.org

Check for updates

OPEN ACCESS

Citation: Yang M-J, Yepez VV, Brandon KO, Reblin M, Pidala J, Jim HSL, et al. (2022) A mindfulness-based stress management program for caregivers of allogeneic hematopoietic stem cell transplant (HCT) patients: Protocol for a randomized controlled trial. PLoS ONE 17(4): e0266316. https://doi.org/10.1371/journal.pone.0266316

Editor: Elisa Panada, UNITED KINGDOM

Received: March 2, 2022

Accepted: March 8, 2022

Published: April 1, 2022

Copyright: © 2022 Yang et al. This is an open access article distributed under the terms of the Creative Commons Attribution License, which permits unrestricted use, distribution, and reproduction in any medium, provided the original author and source are credited.

Funding: The research reported in this publication was supported by an award from the National Cancer Institute (R01CA255265, PI: CV) and by the Participant Research, Interventions, and Measurement Core, and the Cancer Center Support Grant (P30CA076292) at the H. Lee Moffitt Cancer Center & Research Institute, a comprehensive cancer center designated by the National Cancer Institute. MJY was supported by a National Cancer Institute training grant (T32CA090314). URL of each funder website: https://www.cancer.gov/ https://cancercenters.cancer.gov/ The funders had

Abstract

Objectives

Caregivers of allogeneic hematopoietic stem cell transplant (HCT) cancer patients experience high caregiver burden and carry a significant amount of responsibility. Mindfulness has the potential to lessen caregiver burden by aiding in stress management. To date, no studies have examined the efficacy of mindfulness in reducing caregiver burden in this population. Based on our pilot study demonstrating initial feasibility and acceptability of FOCUS (Focusing On mindfulness for Caregivers Under Stress), this 3-arm randomized controlled trial aims to examine the efficacy of a 6-week mindfulness-based stress management program for allogeneic HCT caregivers. Hypotheses include that the FOCUS condition will have lower post-treatment caregiver burden and that patients of these caregivers will have better patient health outcomes compared to other treatment conditions.

Method

Eligible caregivers will be randomly assigned to one of three treatment conditions: FOCUS, Healthy Living (HL; active control), and Enhanced Care (EC; usual care). Caregivers in FOCUS and HL will participate in 6-week weekly individual treatment sessions and will be sent brief daily momentary interventions/messages. Caregivers in all conditions will complete daily diaries over the course of treatment. Patients of enrolled caregivers will be

and will not have a role in study design, data collection and analysis, decision to publish, or preparation of the manuscript.

Competing interests: I have read the journal's policy and the authors of this manuscript have the following competing interests: H.J. is consulting to Janssen Scientific Affairs and Merck and obtained grant funding from Kite Pharma.

enrolled for assessments only. Participants will complete assessments at baseline, end of treatment, 2- and 6-months post-treatment. Biomarker data will be collected via hair cortisol concentrations from caregivers at baseline and 6 months post-treatment.

Results

Recruitment is ongoing.

Conclusions

The data collected from this study will provide evidence on the efficacy of mindfulness in alleviating HCT caregiver stress and impacting patient health outcomes.

Trial registration

The current study is registered in clinicaltrials.gov (NCT05078229); see https://clinicaltrials.gov/ct2/show/NCT05078229?term=christine+vinci&draw=2&rank=1.

Introduction

Allogeneic hematopoietic stem cell transplants (HCT) are considered a possibly curative treatment for hematologic malignancies and disorders (e.g., leukemia, non-Hodgkin lymphoma). Given the extremely intensive nature of the HCT, a full-time caregiver is required [1] who carries a significant responsibility [2,3]. For example, for at least the first 100 days post-transplant, caregivers are responsible for medication administration, arranging patient appointments, food preparation, extensive hygienic precautions, and identifying any early symptoms of infection while managing their own responsibilities (e.g., work, childcare). In particular, allogeneic HCT caregivers experience higher levels of anxiety and stress pre-transplant, as compared to the general population [4], which continues through post-transplant [5]. Such caregiver burden and distress can have an adverse impact on patient health outcomes, including increased anxiety and depression [6], increased healthcare utilization (e.g., emergency department use, [7]) and decreased patient survival [8]. Interventions that alleviate caregiver burden are needed not only to improve caregiver stress but also to have a positive impact on patient health.

Nevertheless, caregiver resources are typically limited to support groups and general stress management services available at the hospital, posing logistical challenges for caregiver attendance, particularly post-discharge. To the best of our knowledge, only one randomized controlled trial (RCT) tested the efficacy of a behavioral intervention using a cognitive-behavioral treatment (CBT) approach among caregivers of only allogeneic HCT patients [9]. This intervention was only delivered post-transplant, after the caregiver was likely experiencing ongoing distress [9]. Further, challenges arose regarding caregiver engagement in all aspects of the intervention and the need for flexibility in scheduling treatment sessions [10]. A second study examined the feasibility of a CBT-based intervention among caregivers of both allogeneic and autologous HCT patients pre- and post-transplant with some flexibility in treatment delivery [11]. Results indicated the intervention was feasible, but the small sample size limited a full examination of treatment outcomes [11]. In sum, there is a need for novel intervention approaches tailored for the allogeneic HCT caregiver population with high flexibility in treatment modality assessing both caregiver and patient outcomes, and that provide support throughout all stages of the caregiving process (pre-transplant through discharge).

Mindfulness-based interventions (MBIs) have the potential to impact the allogeneic HCT caregivers' psychological outcomes. Mindfulness is theorized to target increased awareness of thoughts, emotions, and sensations, with an emphasis on cultivating a nonjudgmental stance to present moment experience. Ultimately, MBIs strive to aid in the development of directed, flexible cognitive processing to observe one's current experience with a sense of acceptance as opposed to trying to change or react to it [12–15]. Based on the extant theories of mindfulness [16–20], the mechanisms that might alter caregiver burden and distress include (1) enhanced awareness of physical sensations and ongoing cognitive-affective processes, (2) increased cognitive flexibility that may allow a caregiver to try a new coping strategy, (3) improved emotion regulation, and (4) shifting in perspectives (i.e., decentering) from distressing thoughts to increased acceptance of the present moment. To expand upon this final point, when caregivers can make meaning of the experience and focus on areas of relational or personal growth, caregiving can be a positive experience [21,22]. Thus, via these proposed mechanisms and through altering the way caregivers respond to stress, MBIs have the potential to lessen caregiver burden.

Currently, a few small-scale, single-arm studies have investigated MBIs for cancer patients and caregivers [23–26]. However, our team's pilot study of FOCUS (Focusing On mindfulness for Caregivers Under Stress) remains the only study that developed and tested the acceptability and feasibility of an MBI for an allogeneic HCT caregiver population with promising results [27]. Importantly, FOCUS was systematically developed with feedback from allogeneic HCT caregivers [28,29].

To fully test the efficacy of FOCUS, we will recruit caregivers of allogeneic HCT patients in the weeks prior to patient transplant and randomize caregivers to one of three-treatment conditions: FOCUS vs Healthy Living (HL) vs Enhanced Care (EC). Caregivers in the FOCUS and HL conditions will attend weekly 45–60 minute individual sessions for 6 consecutive weeks and receive daily brief interventions using a study-specific smartphone application (study app). Caregivers in the EC condition will receive existing usual care for caregivers. The primary hypothesis is that caregivers in FOCUS will report greater reduction in caregiver burden 2 months post-treatment compared to those in HL or EC. The secondary hypothesis is that the patient distress 2 months post-treatment will be lower among patients with caregivers in FOCUS compared to those in HL or EC. As an exploratory hypothesis, we will examine if patient healthcare utilization is lower 2 months and from 3 to 6 months post-treatment among patients with caregivers in FOCUS compared to HL or EC. Potential mechanisms and moderators of treatment on caregiver outcomes will also be explored.

Method

Participants and recruitment

We aim to recruit 270 caregivers and their associated patients on the Blood and Marrow Transplantation (BMT) unit at Moffitt Cancer Center. Potential participants will be identified through a weekly review of an admission list of patients receiving allogeneic HCTs within the next 7–10 days. The current study was approved by the Institutional Review Board (IRB) Advarra, which is used by Moffitt Cancer Center. The protocol and other relevant study documents were approved by the Advarra Institutional Review Board (IRB) which is used by Moffitt Cancer Center. Eligibility criteria of caregiver and patient are presented in Table 1. Recruitment is ongoing. This study received funding in April 2021 and began recruitment in September 2021.

Initial screening

Fig 1 provides an overview of the study timeline. Upon receipt of the patient admission list, study staff will review the medical chart to determine initial eligibility (e.g., cancer diagnosis,

Table 1. Eligibility criteria.

Inclusion Criteria for Caregivers	Inclusion Criteria for Patients
• ≥ 21 years of age<br>• Be the primary caregiver for a patient scheduled to receive an allogeneic HCT at Moffitt Cancer Center<br>• Intend to remain the primary caregiver (i.e., will be the caregiver the majority of the time) throughout patient treatment<br>• Be able to provide informed consent<br>• Be able to read, speak, and write in English<br>• Own a smartphone and be willing to download a study app	• ≥ 21 years of age<br>• Be scheduled to receive an allogeneic HCT at Moffitt Cancer Center<br>• Have a confirmed cancer diagnosis<br>• Be able to provide informed consent<br>• Be able to speak, read, and write in English

https://doi.org/10.1371/journal.pone.0266316.t001

allogeneic transplant, caregiver plan with a primary caregiver). Caregivers of preliminarily eligible patients will be contacted by phone and informed of the goals of the study and all eligibility criteria will be assessed/confirmed. If deemed eligible and the caregiver expresses interest, caregivers will be provided with additional study details and asked to provide informed consent, complete baseline assessments, and provide a hair sample. Patients of caregivers will then be contacted to determine interest and eligibility.

Consent and baseline

Verbal informed consent will be obtained separately for the caregiver and patient. All procedures were approved by Advarra IRB, including verbal informed consent. Both verbal consent and name of the staff who obtained the consent will be documented on the REDCap. Both caregiver and patient will be provided with a copy of the consent form. If the caregiver consents, then the patient will be invited to complete consent. At an upcoming patient medical appointment, caregivers will be asked to provide a small hair sample (approximately 10-30mg) from the posterior vertex of the scalp to test for cortisol levels as an initial index of chronic adrenocortical activity and a biomarker of physiological stress. A hair-care questionnaire will be completed by the caregiver to gather medical information which may interfere with the cortisol readings (e.g., lists of medications, use of steroids, hair-care questions). Both the patient and caregiver will be asked to complete a baseline survey. The caregiver will be told which condition they are randomized to and that the six upcoming treatment sessions will be scheduled for those randomized to FOCUS or HL.

All caregivers, regardless of condition, will be asked to download a study app on their smartphones. Study staff will ensure the caregivers can download the app and the facilitator will instruct them on how to use the app during the first FOCUS or HL session. Staff will instruct those in the EC condition on use of the app following app download. Finally, all caregivers will receive a program packet that consists of a treatment booklet, headphones (for use with meditations for FOCUS or remote sessions for HL), and a list of support groups/resources at Moffitt Cancer Center. In terms of the treatment booklet, a FOCUS booklet will be provided to FOCUS caregivers, and the HL booklet will be distributed to both HL and EC caregivers.

Randomization

Caregiver/patient dyads will be randomized to one of the three treatments using a balanced-permuted block size of six, assigning 90 dyads to each condition. Caregivers will be stratified by gender prior to randomization as research indicates male and female cancer caregivers differ on distress and coping styles [30]. Computer-generated allocation sequence will be used. Study statistician (LRG) will generate the allocation sequence and study staff will enroll and assign participants to intervention conditions. No procedure on blinding or concealment of treatment allocation will be necessary.

	TRANSPLANT TIMELINE							
	Enrollment	Randomization	Admission	Transplant	Post-Discharge		Follow-Up	
DAYS	*-14*	*-10*	*-7*	*0*	*+15*	*+36*	*+92*	*+260*
**ENROLLMENT:**	X							
**Eligibility screen**	X							
**Informed consent**		X						
**Randomization**		X						
**INTERVENTIONS:**								
**FOCUS**			◆———	———	———	———◆		
**HL**			◆———	———	———	———◆		
**EC**			◆———	———	———	———◆		
**ASSESSMENTS[a]:**								
**Caregiver/Patient Demographics**		X						
**Caregiver self-report measures**		X				X	X	X
**Caregiver hair collection for cortisol analysis**		X						X
**Caregiver treatment feedback**		X				X		
**Patient self-report measures**		X				X	X	X
**Patient Interview**		X					X	
**Patient Clinical Information**		X				X	X	X

Fig 1. Schedule of enrollment, interventions, and assessments. Note. [a]See Table 4 for the details. FOCUS = Mindfulness Treatment; HL = Healthy Living Treatment; EC = Enhanced Care.

https://doi.org/10.1371/journal.pone.0266316.g001

Treatment visit: Overview

Fig 1 provides an overview of the timeline for treatment. Both FOCUS and HL caregivers will individually meet with a facilitator (in-person and/or Zoom videoconferencing) weekly for 6 consecutive weeks following enrollment into the study. They will also receive 1 to 3 daily brief momentary interventions/messages through the study app. Session 1 will be designed to be

completed the week prior to transplant, session 2 the week of transplant, and session 3 the first week post-transplant. Thus, sessions 1–3 should occur in person while the patient is staying on the BMT unit. Sessions 4–6 will occur post-discharge once the patient is released within a 30-minute radius of the cancer center; these sessions will be conducted through Zoom video-conferencing. Due to the COVID-19 pandemic, in-person sessions may be modified to be delivered via videoconferencing as needed. At the end of session 1, the facilitator will assist in initiating the study app and describing its functions. All sessions will be audio-recorded for treatment fidelity purposes.

FOCUS condition. The session content of FOCUS is presented in Table 2. FOCUS was systematically developed and pilot tested with feedback from allogeneic HCT caregivers [27–29]. Table 2 provides an overview of session content. Briefly, each session will include 2–3 formal mindfulness meditations led by the facilitator that last approximately 10 minutes each. The first few sessions will be centered on how to direct attention to the breath or some object of attention (e.g., parts of the body). As the sessions progress, caregivers will be asked to apply

Table 2. FOCUS session content.

Session 1 –Mindfulness for Caregivers
• Orientation to treatment<br>• Existing coping skills<br>• Raisin exercise<br>• Introduction to mindfulness<br>• Mindful stretching<br>• Formal vs informal mindfulness<br>• Sitting meditation on the breath
Session 2 –Awareness of Stressors and the Experience of Stress
• Sitting meditation on the breath<br>• Common challenges in meditation<br>• Stress and the body<br>• Body scan meditation<br>• Surfing the stress
Session 3 –Skillful Action
• Sitting meditation on the senses<br>• Uncontrollability vs skillful action<br>• STOP skill (Stop, Take a breath, Observe, Proceed)<br>• Mountain meditation<br>• Preparing for discharge
Session 4 –Thoughts are Thoughts
• Present moment awareness meditation<br>• Discussion on what are thoughts?<br>• Sitting meditation on thoughts<br>• Awareness of thoughts related to emotion and body<br>• Walking meditation
Session 5 –Self-Care and Balance
• Gratitude practice meditation<br>• Daily activities exercise and self-care<br>• Loving kindness meditation<br>• Discussion on the momentary nature of experience
Session 6 –Planning for the Future
• Meditation (caregiver's choice)<br>• Discussion on how to apply what was learned in the treatment when encountering changes in patient health<br>• Discussion on the regular practice of mindfulness<br>• Sitting meditation on the breath

Note. Each session starts with a mindfulness meditation followed by reviewing the home practice from the previous week (sessions 2–6) and concludes with reviewing the home practice for the upcoming week (sessions 1–5).

https://doi.org/10.1371/journal.pone.0266316.t002

these skills to thoughts and emotions. Each week, home practices will be assigned which include exercises relevant to each session theme and the practice of formal meditations 2 times per day (audio recordings of meditations available on the app). At the end of session 3, caregivers will be asked to consider how to prepare for the discharge of the patient, as this is the usual timeframe for most patients to be discharged (this content can be moved if patient discharge is delayed).

During the 6 weeks of treatment, caregivers will interact with the study app in two ways: (1) Receive brief momentary interventions 1–3 times per day, and (2) Listen to the audio-recordings of meditations. A daily meditation reminder with a link to audio-recorded meditations will be deployed at 9am every day. However, caregivers will be able to choose to practice the meditation any time throughout the day by accessing the audio-recordings within the app itself. The brief momentary interventions will consist of brief mindfulness strategies and motivational messages. Brief mindfulness strategies will be randomly drawn from a pool of 96 potential strategies in the following content areas: focusing on the breath, noticing thoughts, awareness of sensations, motivational messages, acceptance/non-judgement, gratitude, loving-kindness, and uncertainty. These strategies aim to enhance and reinforce the use of mindfulness in daily life and should take 1–3 minutes to complete. Examples include: "Over the next several moments, notice any thoughts that come into your mind"; "Allow yourself a couple of minutes to just focus on your breathing in this moment"; "Not knowing sometimes causes distress. Observe this experience with curiosity and notice how it changes over time." Upon completion, caregivers will have the ability to rate the helpfulness of each strategy (1–5 rating scale).

HL condition. HL will match FOCUS in time and provider contact. The treatment content will be based on the American Cancer Society's Caregiver Resource Guide [31] with a specific focus on two sections: Cancer Information and Caregiver Self-Care. A booklet was created by the study team including these two topics, divided into 6 sessions (Table 3). The aim of HL is not to teach active coping strategies to manage stress but to provide information on general topics of self-care commonly known and available to the public. Thus, it is not expected that the HL intervention will have a direct or indirect impact on primary outcomes (e.g., burden) or active mechanisms (e.g., reduced stress).

To match FOCUS on technology interaction, HL caregivers will also interact with the study app in two different ways: (1) Receive brief messages that remind them about the session content, along with motivational messages (1–3 times per day), and (2) Have access to HL booklet content in the form of pdfs within the app. The brief messages will be randomly drawn from a pool of 96 messages in the following content areas: overview of cancer, eating well, financial wellness, sleep, cancer prevention, exercise, and motivational messages. Examples include: "Try to include a fruit and vegetable at every meal"; "If you decide to take a nap, try to take it earlier in the day"; "Staying active can help reduce your stress." Upon completion, caregivers will have the ability to rate the helpfulness of each message (1–5 rating scale).

EC condition. Caregivers in the EC condition will receive treatment that is consistent with what is already offered to all caregivers of allogeneic HCT patients, including the option to attend weekly support groups and meeting with social workers as needed. A handout designed by the study team listing all hospital resources will be provided to those in EC at the consenting session. EC caregivers will also be provided with the same booklet given to HL caregivers. This booklet can be a stand-alone guide for self-care, and so it was provided to those in EC as an additional resource. Caregivers in the EC conditions will not receive any daily momentary interventions/messages or weekly individual sessions.

Study application. LifeData RealLife Exp mobile application (lifedatacorp.com) will be used to deploy daily diaries (all conditions), brief momentary interventions/messages (FOCUS

Table 3. HL session content.

Session 1 –Overview of Cancer
• Orientation to treatment<br>• What is cancer?<br>• Causes of cancer<br>• Pain and physical changes in patients
Session 2 –Eating Well
• Maintaining healthy weight<br>• Fruits and vegetables<br>• Meal planning
Session 3 –Financial Wellness
• Cancer and finances<br>• What do I need to know about my loved one's health insurance?<br>• Common legal issues
Session 4 –Sleep
• Sleep hygiene<br>• Napping<br>• Preparing for bedtime and what to do while in bed
Session 5 –Cancer Prevention
• Limiting alcohol use<br>• Smoking cessation<br>• Protecting your skin<br>• Cancer screening
Session 6 –Exercise
• Recommendations for physical activity<br>• Reducing sedentary behavior

Note. Each session will start with reviewing the home practice from the previous week (sessions 2–6) and concludes with reviewing the home practice for the upcoming week (sessions 1–5).

https://doi.org/10.1371/journal.pone.0266316.t003

and HL only), daily meditations (FOCUS only), and on-demand treatment content corresponding to each treatment condition (FOCUS and HL only; daily meditations for FOCUS and an electronic copy of HL booklet for HL).

Retention procedures

The following procedures will be conducted to reduce attrition: reminder phone calls; flexible scheduling of caregiver treatment sessions to accommodate different schedules; meeting with the caregiver for sessions 1–3 on the transplant unit (or via videoconferencing); meeting for sessions 4–6 via videoconferencing; and requiring a functioning phone number and home address to contact caregivers by phone or mail as needed. Greeting cards will be also mailed at key points throughout the study (e.g., patient discharge, holidays, birthdays). Caregivers and patients will be financially compensated for completing the assessment measures. Caregivers will receive $25 for completing the surveys at baseline, end of treatment, and 2-month follow-up. For completing the 6-month follow up survey, the caregiver will be compensated $50. If the caregiver completes at least 70% of the daily diary questions, they will receive a bonus payment of $40 at the end of treatment. The patient will be compensated $20 at each assessment time point.

Follow-up assessment visits

Caregiver/patient dyads will complete the follow-up survey at 2-month and 6-months post-caregiver treatment. Hair samples will be collected at the 6-month assessment from caregivers.

All follow-up assessments will be sent by text and/or email via an electronic link. If requested, paper surveys will be mailed to caregivers.

Measures

Table 4 provides an overview of visit measures and measures on patient health care utilization. A summary is provided below. Questionnaire data will be collected via REDCap.

Demographics and caregiver/patient characteristics. Demographics will include gender, age, ethnicity, race, education, employment, income, partner status, and insurance status. Additional questions for the caregiver will include relationship to patient and proximity of home residence to the cancer center. Attendance at any support groups or other psychological treatment received by caregivers and patients will also be collected. Clinical characteristics (e.g., cancer type, treatment) will be assessed by chart abstraction.

Caregiver burden and distress. The primary caregiver outcome, burden, will be assessed using a validated self-report measure [32]. Additionally, depression [33], anxiety [35], post traumatic growth [36], and overall distress [34] will be measured.

Patient distress and health care utilization. Patient depression, anxiety, and overall distress will be measured using the same questionnaires the caregivers complete. Healthcare utilization will be assessed by extracting the following from patient charts: readmissions to the hospital, length of stay of all hospital readmissions, and unexpected clinic visits post-discharge.

Additional caregiver variables. Stress and affect will be captured by self-report and biomarker data (hair cortisol). Specifically, perceived stress [38], trauma symptoms around the diagnosis of cancer [39,48], and affect [37] will be measured. Hair samples will be collected at baseline and the 6-month follow-up for cortisol assay using previously validated methods [49]. Sample will be cut to a standard length of 3cm before processing, thereby providing an integrated measure of cortisol production during the prior 3 months based on an average hair growth rate of 1cm per month [49–51]. Mindfulness will be measured via two measures [40,41]. Self-efficacy in the context of transplant [42], perceived social support [43], and mindful self-care [44] will be also assessed.

Treatment feedback. At the end of treatment (i.e., after the caregiver completes their treatment), a battery of questionnaires to obtain treatment feedback will be administered. We will use a validated self-report measure to assess treatment satisfaction [46] and questionnaires developed by the study team will assess coping strategies. Among FOCUS and HL caregivers, perceived utility of the treatment and study app, the intent to use the skills learned, preferences for and benefits of the skills learned, appropriateness of the brief momentary interventions/ messages, and barriers to treatment will be assessed. Working alliance with the facilitator [47] and preference for each meditation (FOCUS only) or session topic (HL only) using team-developed questionnaires will be also measured.

Additional questionnaires to assess caregiver/patient behaviors. Among all caregivers, additional questionnaires will be collected to assess factors that might impact caregivers' behaviors including tobacco and alcohol use [45]. Questionnaires on the impact of COVID-19 on distress level/interaction between dyads and mental health service utilization will be collected from both caregivers and patients. Additionally, a patient interview will be developed by the team that comprises open-ended questions to assess thoughts, feelings, and coping during the pre-hospitalization, transplant/hospitalization, and post-hospitalization periods, as well as general questions about stress management needs. Approximately 15 patients each from the HL and FOCUS conditions will be asked to complete the interview with a staff member by telephone. This information will be used to inform any future treatment development that could include a patient component.

Table 4. Overview of measures.

Survey	Timeframe			
**Measure**	**Baseline**	**EOT**	**2 Month Follow-up**	**6 Month Follow-up**
**CAREGIVER MEASURES**				
Demographics/Use of Mental Health Services				
Demographics	X			
Mental Health Services	X	X	X	X
Burden/Distress/Affect/Stress				
The Zarit Burden Interview [32]	X	X	X	X
Center for Epidemiological Studies Depression Scale [33]	X	X	X	X
Distress Thermometer [34]	X	X	X	X
Generalized Anxiety Disorder-7 [35]	X	X	X	X
Post-Traumatic Growth Inventory [36]	X	X	X	X
Positive and Negative Affect Scale [37]	X	X	X	X
Perceived Stress Scale [38]	X	X	X	X
Impact of Event Scale [39]	X	X	X	X
Hair collection for cortisol analysis	X			X
Mindfulness/Self-Efficacy/Support/Self-Care				
Mindful Attention Awareness Scale [40]	X	X	X	X
Five Facet Mindfulness Questionnaire [41]	X	X	X	X
Caregiver Self-Efficacy Scale [42]	X	X	X	X
Functional Social Support Questionnaire [43]	X	X	X	X
Mindful Self-Care [44]	X	X	X	X
Additional Caregiver Variables				
Impact of COVID-19	X	X	X	X
Tobacco Use (Quantity/Frequency)	X	X	X	X
Alcohol Use Disorders Identification Test-C [45]	X	X	X	X
Coping	X	X	X	X
Feedback from Participants				
Client Satisfaction Questionnaire [46]		X		
Working Alliance Inventory-Short Revised [47] (FOCUS & HL)		X		
Coping Strategies (FOCUS & HL)		X	X[a]	X[a]
Utility of Treatment and Study App		X		
**PATIENT MEASURES**				
Demographics/Use of Mental Health Services				
Demographics	X			
Mental Health Services	X	X	X	X
Distress				
Center for Epidemiological Studies Depression Scale [33]	X	X	X	X
Distress Thermometer [34]	X	X	X	X
Generalized Anxiety Disorder-7 [35]	X	X	X	X
Additional Patient Variables				
Impact of COVID-19	X	X	X	X
Patient Interview			X	
Patient Information/Patient Healthcare Utilization (abstracted from medical chart)				
Clinical Characteristics	X			
Readmissions		X	X	X
Readmission Length		X	X	X

(*Continued*)

Table 4. (Continued)

Survey	Timeframe			
**Measure**	**Baseline**	**EOT**	**2 Month Follow-up**	**6 Month Follow-up**
Unexpected Clinic Visits		X	X	X

Note. [a] Completed only in FOCUS. EOT = End of Treatment. Measures without citations refer to those developed by team.

https://doi.org/10.1371/journal.pone.0266316.t004

Daily diary on study app. Evening daily diaries will be delivered one time a day via the study app and will consist of 10 questions assessing state mindfulness (attention, nonjudgment/acceptance, decentering), state affect, and self-efficacy. The daily diary will be delivered in the evening at a time selected by the caregiver at the beginning of the study (between 5pm and 8pm). The daily diary will take no longer than 1 minute to complete.

Treatment delivery and fidelity

To ensure treatment delivery follows the protocol precisely and to prevent counselor drift and contamination, facilitator selection criteria, extensive training, ongoing monitoring and supervision of treatment delivery, treatment fidelity coding, and facilitator competence will be planned. Facilitators will concurrently provide both FOCUS and HL treatments to reduce any therapist-specific effects.

Facilitator selection criteria. Facilitators will be required to have either a minimum of a bachelor's degree in counseling, psychology, social work, or a related field, or, have extensive experience/background in the delivery of mindfulness-based practices (e.g., meditation, yoga). All facilitators will be expected to have an ongoing daily mindfulness practice. If not already completed, facilitators will need to complete additional training to deliver the treatments with fidelity: completing a formal mindfulness training (e.g., Mindfulness-Based Stress Reduction), attending a silent meditation retreat, and completing formal training in the delivery of FOCUS and HL.

Supervision and treatment fidelity. A clinical psychologist will lead facilitator training for FOCUS and HL, which will include readings of the FOCUS and HL manuals and practicing and role playing each session of FOCUS and HL. To ensure fidelity, weekly supervision with the opportunity to review audio-recordings of the sessions will be conducted. About 10% of the audio recordings will be assessed for facilitator competence and adherence via a modified version of the validated Mindfulness-Based Relapse Prevention Adherence and Competence Scale [52] for FOCUS; a separate competence and adherence measure will be created for HL.

Treatment adherence. To assess treatment adherence, home practice will be monitored weekly via the study app (e.g., amount of time spent daily for meditations). Facilitators will also complete a session note following each treatment session for each participant.

Data management plan and safety monitoring plan

Data and safety monitoring will be ongoing by the Principal Investigator (PI), Moffitt Cancer Center's Protocol Review and Monitoring Committee, and the IRB. Participant data will be associated with participant study ID and not directly associable with names or other identifying information. Questionnaire/interview data will be collected and recorded via REDCap and will only be identified with the participant's study ID. All data including audio-recordings of the sessions will be stored on an encrypted secure database. Access to data will be restricted to staff who have certificates in the Human Subjects Research at the Collaborative Institutional

Training Initiative. Data accuracy will be subject to random audit. Monthly data management reports will be made by the project data coordinator, including data entry progress, error rates, range checks, and general descriptive statistics. Research staff will report any potential adverse events immediately to the PI. The PI will follow the adverse event reporting guidelines of the IRB. Any IRB actions in relation to this protocol will also be reported to the funding agency. Data will be made available with a reasonable request within one year following the completion of data collection and initiation of data dissemination activities on the main outcome.

Planned analysis

Sample size and power. There will be 90 dyads randomized to each condition, with at least 63 per condition (30% dropout) expected to complete the 2-month assessment. PASS 16 was used to estimate power and effect size based on this sample size. To test the primary hypothesis, for the FOCUS vs HL comparison, with alpha set at .05, a two-sided test, and condition n's of 63, it is estimated that power $>.80$ will detect a standardized difference between means of 0.51, a medium effect size. For the FOCUS vs EC comparison, under the same conditions as above but with alpha = .025, it is estimated that power $>.80$ will detect a standardized difference between means of 0.56, which can be described as slightly greater than a medium effect size. Power estimates for the secondary hypothesis on patient distress and the exploratory hypothesis of healthcare utilization are expected to have fewer participants and therefore the minimum effect sizes will be higher than those for the primary hypothesis (e.g., if condition n's = 50, then effect sizes increase to 0.57 [HL] and 0.63 [EC] and).

Analytic plan. Descriptive statistics will be used to summarize and evaluate the distributions of all study variables. Transformations (e.g., log, square root) will be applied, as needed, prior to hypothesis testing and the Shapiro-Wilks test will be used to evaluate normality. Missing data analyses will be conducted. Independent sample t-tests will be used to identify condition differences, despite randomization, in baseline measures that will then be included as covariates when testing hypotheses. Primary analyses for the primary and secondary hypotheses will use generalized estimating equations (GEE) with an identity link function, linear regression, and compound symmetry for the working correlation matrix. The Holm method [53] will be applied to control for experiment-wise error in the primary analyses, with alpha set at .025 for tests of FOCUS vs EC and set at .05 for FOCUS vs HL.

To examine the primary hypothesis, the primary predictors of the GEE model will be condition (e.g., FOCUS vs EC), time (end of treatment; 8 weeks vs 2 months), and their interaction. The GEE will include as a covariate any baseline measure that differs by condition ($p < .05$) as a potential confound (e.g., age, relationship to patient, baseline burden). Missing data in the response variable will be handled using inverse probability subject specific weighting methods implemented in SAS PROC GEE. If baseline variables are significantly related to missingness of the response variable, then full information maximum likelihood or multiple imputation will be used to handle missing data. The hypothesis will be tested using a planned contrast of condition at 2 months assessing the prediction that FOCUS will have a lower mean burden than the comparison condition (HL or EC).

Discussion

Studies that examine the efficacy of stress reduction for caregivers are limited. To the best of our knowledge, our team's pilot study is the first that reported on the feasibility and acceptability of an MBI among allogeneic HCT caregivers [27]. As an extension of our team's prior work, the current manuscript describes the rationale and design of an NIH-funded, 3-arm RCT that aims to recruit caregivers of allogeneic HCT cancer patients (N = 270) and their

patients with the overall goal of testing the efficacy of a 6-week mindfulness-based stress management program on reducing caregiver burden, patient distress, and patient health care utilization, as compared to two comparison conditions.

Several strengths of the study design are worth noting. First, the FOCUS intervention was systematically developed based on feedback from allogeneic HCT caregivers and HCT clinic staff, and extant literature on mindfulness and cancer caregivers more broadly [28,29]. Our pilot study supported its potential to reduce caregiver burden/distress and increase post traumatic growth, as well as the feasibility and acceptability of the current study procedures [27]. Second, the interventions will be provided over the entire course of the transplant process (pre- and post-transplant). Providing intervention content pre-transplant will hopefully aid caregivers in preparing for what to expect and how to cope. Continuing the intervention through the transplant and post-discharge period provides an excellent opportunity to apply what was learned in earlier sessions while the caregiver is in the role of taking care of the patient full-time. Third, the flexibility in scheduling and treatment delivery modality (in-person or videoconferencing) reduces caregiver burden and potentially maximizes treatment engagement. This should enhance future scalability to other health care settings regardless of unexpected events (e.g., COVID-19). Fourth, brief momentary interventions/messages supplement the main intervention providing an opportunity to apply learned skills to daily life. Fifth, collecting biomarker and daily diary data will allow a thorough investigation of proposed mechanisms. Sixth, if the primary hypothesis is supported, the results will provide strong evidence on the potential of an MBI for other cancer caregiver populations where caregiver burden and responsibilities are high (e.g., head and neck). Alternatively, if FOCUS does not demonstrate superior outcomes compared to HL, we will know that an intervention such as HL that requires fewer resources may be sufficient to reduce caregiver burden.

Limitations should also be discussed. All caregivers are asked to complete daily diaries and those in FOCUS and HL will additionally complete aspects of the intervention in the study app, which may increase participant burden. To reduce burden, the daily diary was designed to take less than 1 minute to complete. All aspects of the app were also designed with the ability to delay responding and/or to not respond if unable to do so. It is also unclear what impact the COVID-19 pandemic will have on study results. We have included questions to assess COVID-related distress at the end of the study.

This project is the first full-scale RCT to evaluate the efficacy of an MBI for allogeneic HCT caregiver burden that also includes assessing patient distress and healthcare utilization. Other unique aspects of the study include the collection of biomarker data, incorporation of brief momentary interventions/messages, and a daily diary component to collect mechanistic data over the course of treatment. Study results will inform the next line research including dissemination and implementation within HCT clinics across different cancer centers, and its integration in the clinic workflow with provider training.

Supporting information

S1 Checklist. SPIRIT_Fillable-checklist-15-Aug-2013.
(DOC)

S1 File. MCC20786_Protocol v5_1_6_2022 clean.
(PDF)

Author Contributions

Conceptualization: Christine Vinci.

Funding acquisition: Christine Vinci.

Methodology: Maija Reblin, Joseph Pidala, Heather S. L. Jim, Jerrold S. Meyer, L. Robert Gore, Nandita Khera, Christine Vinci.

Writing – original draft: Min-Jeong Yang.

Writing – review & editing: Valerie V. Yepez, Karen O. Brandon, Maija Reblin, Joseph Pidala, Heather S. L. Jim, Jerrold S. Meyer, L. Robert Gore, Nandita Khera, Penny Lau, Rachel M. Sauls, Sarah R. Jones, Christine Vinci.

References

1. Gyurkocza B., Rezvani A., and Storb R.F., Allogeneic hematopoietic cell transplantation: the state of the art. Expert Rev Hematol, 2010. 3(3): p. 285–299. https://doi.org/10.1586/ehm.10.21 PMID: 20871781
2. Gemmill R., et al., Informal caregivers of hematopoietic cell transplant patients: a review and recommendations for interventions and research. Cancer Nurs, 2011. 34(6): p. E13. https://doi.org/10.1097/NCC.0b013e31820a592d PMID: 21242762
3. Wulff-Burchfield E., Jagasia M., and Savani B., Long-term follow-up of informal caregivers after allo-SCT: a systematic review. Bone Marrow Transplant, 2013. 48(4): p. 469. https://doi.org/10.1038/bmt.2012.123 PMID: 22732697
4. Simoneau T.L., et al., Elevated peri-transplant distress in caregivers of allogeneic blood or marrow transplant patients. Psychooncology, 2013. 22(9): p. 2064–2070. https://doi.org/10.1002/pon.3259 PMID: 23440998
5. Jim H.S., et al., Patient education in allogeneic hematopoietic cell transplant: what patients wish they had known about quality of life. Bone Marrow Transplant, 2014. 49(2): p. 299–303. https://doi.org/10.1038/bmt.2013.158 PMID: 24121210
6. Jacobs J.M., et al., Distress is interdependent in patients and caregivers with newly diagnosed incurable cancers. Ann Behav Med, 2017. 51(4): p. 519–531. https://doi.org/10.1007/s12160-017-9875-3 PMID: 28097515
7. Ankuda C.K., et al., Association between spousal caregiver well-being and care recipient healthcare expenditures. J Am Geriatr Soc, 2017. 65(10): p. 2220–2226. https://doi.org/10.1111/jgs.15039 PMID: 28836269
8. Dionne-Odom J.N., et al., Associations between advanced cancer patients' survival and family caregiver presence and burden. Cancer Med, 2016. 5(5): p. 853–862. https://doi.org/10.1002/cam4.653 PMID: 26860217
9. Laudenslager M.L., et al., A randomized control trial of a psychosocial intervention for caregivers of allogeneic hematopoietic stem cell transplant patients: effects on distress. Bone Marrow Transplant, 2015. 50(8): p. 1110–1118. https://doi.org/10.1038/bmt.2015.104 PMID: 25961767
10. Simoneau T.L., et al., An evidence-based stress management intervention for allogeneic hematopoietic stem cell transplant caregivers: development, feasibility and acceptability. Support Care Cancer, 2017. https://doi.org/10.1007/s00520-017-3660-5 PMID: 28283805
11. El-Jawahri A., et al., Multimodal psychosocial intervention for family caregivers of patients undergoing hematopoietic stem cell transplantation: A randomized clinical trial. Cancer, 2020. 126(8): p. 1758–1765. https://doi.org/10.1002/cncr.32680 PMID: 31899552
12. Kabat-Zinn J., Full catastrophe living: Using the wisdom of your body and mind to face stress, pain, and illness. 1990: Random House LLC. PMID: 2383411
13. Kabat-Zinn J., Wherever you go, there you are: Mindfulness in everyday life. 1994, New York, NY: Hyperion.
14. Teasdale J.D., Segal Z., and Williams J.M.G., How does cognitive therapy prevent depressive relapse and why should attentional control (mindfulness) training help? Behav Res Ther, 1995. 33(1): p. 25–39. https://doi.org/10.1016/0005-7967(94)e0011-7 PMID: 7872934
15. Wood A.W., Gonzalez J., and Barden S.M., Mindful caring: using mindfulness-based cognitive therapy with caregivers of cancer survivors. J Psychosoc Oncol, 2015. 33(1): p. 66–84. https://doi.org/10.1080/07347332.2014.977418 PMID: 25397963
16. Creswell J.D. and Lindsay E.K., How does mindfulness training affect health? A mindfulness stress buffering account. Curr Dir Psychol Sci, 2014. 23(6): p. 401–407.
17. Carmody J., et al., An empirical study of the mechanisms of mindfulness in a mindfulness-based stress reduction program. J Clin Psychol, 2009. 65(6): p. 613–626. https://doi.org/10.1002/jclp.20579 PMID: 19267330

18. Grabovac A.D., Lau M.A., and Willett B.R., Mechanisms of mindfulness: A Buddhist psychological model. Mindfulness, 2011. 2(3): p. 154–166.

19. Hölzel B.K., et al., How does mindfulness meditation work? Proposing mechanisms of action from a conceptual and neural perspective. Perspect Psychol Sci, 2011. 6(6): p. 537–559. https://doi.org/10.1177/1745691611419671 PMID: 26168376

20. Tang Y.-Y., Hölzel B.K., and Posner M.I., The neuroscience of mindfulness meditation. Nat Rev Neurosci, 2015. 16(4): p. 213–225. https://doi.org/10.1038/nrn3916 PMID: 25783612

21. Kim Y., Schulz R., and Carver C.S., Benefit finding in the cancer caregiving experience. Psychosom Med, 2007. 69(3): p. 283–291. https://doi.org/10.1097/PSY.0b013e3180417cf4 PMID: 17420443

22. Li Q. and Loke A.Y., The positive aspects of caregiving for cancer patients: a critical review of the literature and directions for future research. Psychooncology, 2013. 22(11): p. 2399–2407. https://doi.org/10.1002/pon.3311 PMID: 23712938

23. Birnie K., Garland S.N., and Carlson L.E., Psychological benefits for cancer patients and their partners participating in mindfulness-based stress reduction (MBSR). Psychooncology, 2010. 19(9): p. 1004–1009. https://doi.org/10.1002/pon.1651 PMID: 19918956

24. van den Hurk D.G., et al., Mindfulness-Based Stress Reduction for lung cancer patients and their partners: Results of a mixed methods pilot study. Palliat Med, 2015. 29(7): p. 652–660. https://doi.org/10.1177/0269216315572720 PMID: 25701663

25. Kubo A., et al., A randomized controlled trial of mHealth mindfulness intervention for cancer patients and informal cancer caregivers: a feasibility study within an integrated health care delivery system. Integr Cancer Ther, 2019. 18: p. 1534735419850634. https://doi.org/10.1177/1534735419850634 PMID: 31092044

26. Fish J.A., et al., Mindfulness-based Cancer Stress Management: impact of a mindfulness-based programme on psychological distress and quality of life. Eur J Cancer Care, 2014. 23(3): p. 413–421. https://doi.org/10.1111/ecc.12136 PMID: 24118428

27. Vinci C., et al., A mindfulness-based intervention for caregivers of allogeneic hematopoietic stem cell transplant patients: Pilot results. Psychooncology, 2020. 29(5): p. 934–937. https://doi.org/10.1002/pon.5353 PMID: 32043667

28. Vinci C., et al., Understanding coping strategies of cancer caregivers to inform mindfulness-based interventions: A qualitative study. Eur J Integr Med, 2019. 30: p. 100936.

29. Vinci C., et al., Understanding preferences for a mindfulness-based stress management program among caregivers of hematopoietic cell transplant patients. Complement Ther Clin Pract, 2018. 33: p. 164–169. https://doi.org/10.1016/j.ctcp.2018.10.007 PMID: 30396616

30. Ketcher D., et al., Caring for a spouse with advanced cancer: similarities and differences for male and female caregivers. J Behav Med, 2019: p. 1–12. https://doi.org/10.1007/s10865-019-00128-y PMID: 31845168

31. American Cancer Society, Cargiver Resource Guide: Caring for a Loved One with Cancer. 2019.

32. Bédard M., et al., The Zarit Burden Interview: a new short version and screening version. Gerontologist, 2001. 41(5): p. 652–657. https://doi.org/10.1093/geront/41.5.652 PMID: 11574710

33. Radloff L.S., The CES-D scale: A self-report depression scale for research in the general population. Appl Psychol Meas, 1977. 1(3): p. 385–401.

34. Psychosocial Distress Practice Guidelines Panel, NCCN practice guidelines for the management of psychosocial distress. National Comprehensive Cancer Network. Oncology (Williston Park), 1999. 13 (5a): p. 113–47. PMID: 10370925

35. Spitzer R.L., et al., A brief measure for assessing generalized anxiety disorder: the GAD-7. Arch Intern Med, 2006. 166(10): p. 1092–1097. https://doi.org/10.1001/archinte.166.10.1092 PMID: 16717171

36. Tedeschi R.G. and Calhoun L.G., The Posttraumatic Growth Inventory: Measuring the positive legacy of trauma. J Trauma Stress, 1996. 9(3): p. 455–471. https://doi.org/10.1007/BF02103658 PMID: 8827649

37. Watson D., Clark L.A., and Tellegen A., Development and validation of brief measures of positive and negative affect: the PANAS scales. J Pers Soc Psychol, 1988. 54(6): p. 1063–1070. https://doi.org/10.1037//0022-3514.54.6.1063 PMID: 3397865

38. Cohen S., Kamarck T., and Mermelstein R., A global measure of perceived stress. J Health Soc Behav, 1983: p. 385–396. PMID: 6668417

39. Weiss D.S., The impact of event scale: revised, in Cross-cultural assessment of psychological trauma and PTSD. 2007, Springer. p. 219–238.

40. Brown K.W. and Ryan R.M., The benefits of being present: mindfulness and its role in psychological well-being. J Pers Soc Psychol, 2003. 84(4): p. 822. https://doi.org/10.1037/0022-3514.84.4.822 PMID: 12703651

41. Baer R.A., et al., Using self-report assessment methods to explore facets of mindfulness. Assessment, 2006. 13(1): p. 27–45. https://doi.org/10.1177/1073191105283504 PMID: 16443717

42. Ugalde A., Krishnasamy M., and Schofield P., Development of an instrument to measure self-efficacy in caregivers of people with advanced cancer. Psychooncology, 2013. 22(6): p. 1428–1434. https://doi.org/10.1002/pon.3160 PMID: 22941754

43. Broadhead W., et al., The Duke-UNC Functional Social Support Questionnaire: Measurement of social support in family medicine patients. Med Care, 1988: p. 709–723. https://doi.org/10.1097/00005650-198807000-00006 PMID: 3393031

44. Cook-Cottone C.P. and Guyker W.M., The development and validation of the Mindful Self-Care Scale (MSCS): An assessment of practices that support positive embodiment. Mindfulness, 2018. 9(1): p. 161–175.

45. Bush K., et al., The AUDIT alcohol consumption questions (AUDIT-C): an effective brief screening test for problem drinking. Arch Intern Med, 1998. 158(16): p. 1789–1795. https://doi.org/10.1001/archinte.158.16.1789 PMID: 9738608

46. Larsen D.L., et al., Assessment of client/patient satisfaction: development of a general scale. Eval Program Plann, 1979. 2(3): p. 197–207. https://doi.org/10.1016/0149-7189(79)90094-6 PMID: 10245370

47. Horvath A.O. and Greenberg L.S., Development and validation of the Working Alliance Inventory. J Couns Psychol, 1989. 36(2): p. 223.

48. Creamer M., Bell R., and Failla S., Psychometric properties of the impact of event scale—revised. Beh Res Ther, 2003. 41(12): p. 1489–1496. https://doi.org/10.1016/j.brat.2003.07.010 PMID: 14705607

49. Meyer J., et al., Extraction and analysis of cortisol from human and monkey hair. J Vis Exp, 2014(83): p. e50882. https://doi.org/10.3791/50882 PMID: 24513702

50. Meyer J.S. and Novak M.A., Minireview: hair cortisol: a novel biomarker of hypothalamic-pituitary-adrenocortical activity. Endocrinology, 2012. 153(9): p. 4120–4127. https://doi.org/10.1210/en.2012-1226 PMID: 22778226

51. Greff M.J., et al., Hair cortisol analysis: An update on methodological considerations and clinical applications. Clin Biochem, 2019. 63: p. 1–9. https://doi.org/10.1016/j.clinbiochem.2018.09.010 PMID: 30261181

52. Chawla N., et al., The mindfulness-based relapse prevention adherence and competence scale: development, interrater reliability, and validity. Psychother Res, 2010. 20(4): p. 388–397. https://doi.org/10.1080/10503300903544257 PMID: 20204916

53. Holm S., A simple sequentially rejective multiple test procedure. Scand J Statist, 1979: p. 65–70.
